# Knowledge, attitudes, and practices associated with zoonotic disease transmission risk in North Sulawesi, Indonesia

**DOI:** 10.1186/s42522-022-00067-w

**Published:** 2022-06-03

**Authors:** Tina Kusumaningrum, Alice Latinne, Stephanie Martinez, Jusuf Kalengkongan, Ageng Wiyatno, Aghnianditya Kresno Dewantari, Novie Kasenda, Janno B. B. Bernadus, Ungke Anton Jaya, Chairin Nisa Ma’roef, Leilani Francisco, Emily Hagan, Maureen Miller, Khin Saw Aye Myint, Peter Daszak, Kevin J. Olival, Suryo Saputro, Joko Pamungkas, Dodi Safari

**Affiliations:** 1 Eijkman Institute of Molecular Biology, Jakarta, Indonesia; 2 EcoHealth Alliance, New York, USA; 3 Present address: Wildlife Conservation Society, Viet Nam Country Program, Ha Noi, Viet Nam; 4 Present address: Wildlife Conservation Society, Health Program, Bronx, NY USA; 5 Faculty of Medicine, Sam Ratulangi University, Manado, Indonesia; 6 Present address: Henry M. Jackson Foundation, Bethesda, MD USA; 7 Present address: Mailman School of Public Health, Columbia University, New York, USA; 8 Primate Research Center, IPB University, Bogor, Indonesia; 9 Faculty of Veterinary Medicine, IPB University, Bogor, Indonesia

**Keywords:** Knowledge, Attitude, Practice, Wildlife, Zoonotic, Risk, Indonesia

## Abstract

**Background:**

Hunters, vendors, and consumers are key actors in the wildlife trade value chain in North Sulawesi, Indonesia, and potentially face an elevated risk of exposure to zoonotic diseases. Understanding the knowledge, attitudes, and practices (KAP) associated with the risk of zoonotic disease transmission in these communities is therefore critical for developing recommendations to prevent or mitigate zoonotic outbreaks in the future.

**Methods:**

Qualitative and quantitative methods were combined to understand KAP associated zoonotic diseases transmission risk in communities involved in the wildlife trade in North Sulawesi. Qualitative data were collected through semi-structured ethnographic interviews and focus group discussions (FGDs) while quantitative data were collected using questionnaires. We conducted 46 ethnographic interviews and 2 FGDs in 2016, and 477 questionnaire administrations in 2017–2018 in communities from five districts in North Sulawesi. We also collected biological specimens, including nasal swab, oropharyngeal swab, and blood, from 254 participants. The study sites were targeted based on known wildlife consumption and trade activities. The participants for qualitative data collection were purposively selected while participants for quantitative data collection were randomly selected. Biological samples were tested for five viral families including Coronaviridae, Filoviridae, Flaviviridae, Orthomyxoviridae and Paramyxoviridae.

**Results:**

Knowledge regarding disease transmission from animals to humans was similar across the participants in qualitative focus groups, including knowledge of rabies and bird flu as zoonotic diseases. However, only a small fraction of the participants from the quantitative group (1%) considered that contact with wild animals could cause sickness. Our biological specimen testing identified a single individual (1/254, 0.004%) who was sampled in 2018 with serological evidence of sarbecovirus exposure. Overall, participants were aware of some level of risk in working with open wounds while slaughtering or butchering an animal (71%) but most did not know what the specific risks were. However, significant differences in the attitudes or beliefs around zoonotic disease risk and health seeking behaviors were observed across our study sites in North Sulawesi.

**Conclusions:**

Our study showed variable levels of knowledge, attitudes, and practices associated with the risk of zoonotic disease transmission among study participants. These findings can be used to develop locally responsive recommendations to mitigate zoonotic disease transmission.

**Supplementary Information:**

The online version contains supplementary material available at 10.1186/s42522-022-00067-w.

## Introduction

More than two-thirds of the 260 known human viruses have resulted from zoonotic spillover [1]. In the last two decades, a series of emerging and re-emerging zoonotic viruses have originated from wildlife, especially from Africa and Asia, and were either transmitted directly or indirectly through an intermediate host, to humans [2–5]. The most recent emerging infectious disease, COVID-19, was first detected in Wuhan, China at the end of 2019 and is presumed to have emerged from rhinolophid bats, although more research is needed to understand its spillover [6–9]. Indeed, the risk of future SARS related coronavirus spillover events extends across Southeast Asia where natural reservoir host species and human populations overlap [9].

Indonesia, an archipelago of over 17,000 islands in equatorial Southeast Asia, is considered a ‘megadiverse’ country in terms of animal species richness, and has the highest National Biodiversity Index in the ASEAN region [10]. In the past, the country has suffered the impacts of major zoonotic disease outbreaks, including Highly Pathogenic Avian Influenza (HPAI), which impacted poultry and human populations and caused an economic loss of USD 470 million during the period of 2003 to 2009 [11].

In some parts of Indonesia, wild animal consumption, including bushmeat, is a common cultural practice which necessitates wildlife hunting either for direct consumption or for sale in markets and restaurants [12–16]. In Papua island, studies reported that various species of mammals, birds, and reptiles were hunted to fulfill animal protein needs for the household [16, 17]. Although wildlife hunting and consumption activities occur in several parts of the country, North Sulawesi Province, on the island of Sulawesi, is where wildlife is sold and consumed in great volume. Previous studies have reported that various species such as bats, rodents, snakes, and wild pigs were the most common bushmeat sold and found daily in the markets of the province [15, 16]. Other wildlife such as non-human primates, anoas, cuscuses, and wild boar were occasionally available and sold in the markets, although in smaller quantities [12, 13]. High market demand caused rapid exploitation of wildlife in North Sulawesi and facilitated the establishment of a well-organized wildlife trade network in Sulawesi, with some species occasionally imported from Kalimantan, a neighboring island [12, 13]. This network resulted in widespread exposure of local populations, including hunters, wild meat slaughterers, vendors, and consumers, to wildlife, and increased the risk of zoonotic infection through human-animal interactions [18–20]. Specific activities which bring humans into close contact with wild animals such as hunting and culinary practices determine the intensity of exposure to zoonotic pathogens [21]. These behaviours and cultural practices can significantly contribute to disease spillover and transmission [22, 23]. Risk is further increased as the targeted wildlife species (bats, rodents) are among those harboring the highest proportion of zoonotic viruses, which is positively correlated with the increasing chance of zoonotic spillover [24]. In addition, knowledge and attitudes regarding zoonotic diseases were reported to affect prevalence rates or outbreak occurrence of such diseases [25].

This study was conducted with the aim of understanding the human behaviors, attitudes, and knowledge that may increase or decrease, the risk of spillover of pathogens from animals to humans, and to assess the socio-economic factors influencing those practices among high-risk communities in North Sulawesi Province. Data were collected using qualitative and quantitative approaches through in-depth interviews and focus groups, and questionnaire administration, respectively. Biological samples were also collected from a subset of participants to detect potential zoonotic viruses present in these communities.

## Methods

### Study location, settings, and population

**Fig. 1:**
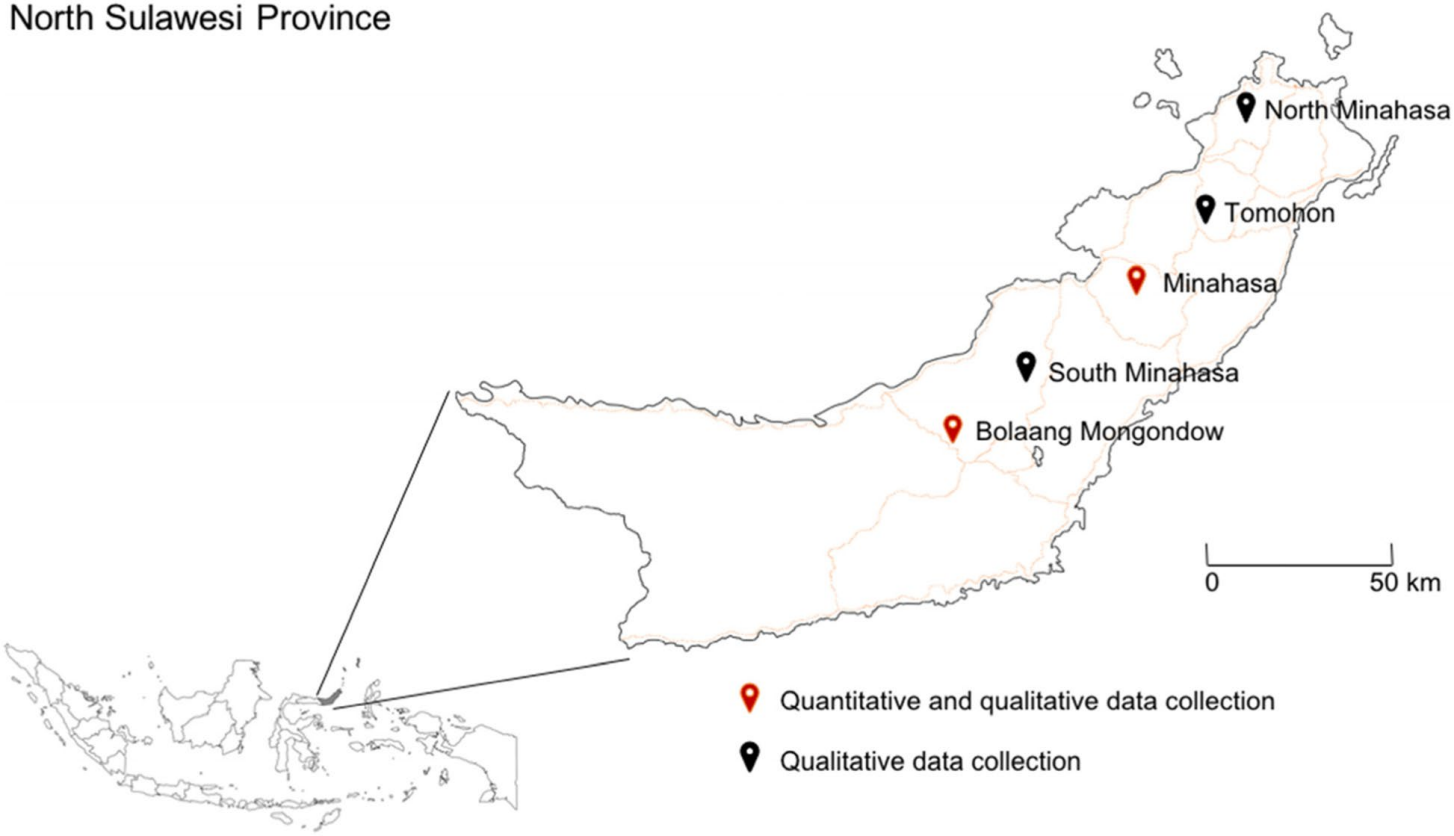
Locations of the five districts in North Sulawesi Province where this study was conducted in communities where wildlife consumption is a common cultural habit

This study is a part of the USAID PREDICT project which investigated the risk of zoonotic disease spillover at human-wildlife interfaces across ~ 30 countries including Indonesia [19]. Sampling locations in Indonesia were selected in areas with known wildlife consumption and trade, including wildlife hunting, bushmeat sale, and butchering, which we considered a high-risk activity for zoonotic spillover. Qualitative interviews and FGD data were collected from March to August 2016 and quantitative data from October 2017 to December 2018. The study was conducted in five districts in North Sulawesi Province: Minahasa, South Minahasa, North Minahasa, Bolaang Mongondow, and Tomohon (Fig. 1). The qualitative data collection was conducted in all five districts: Minahasa (1 village), South Minahasa (4 villages), North Minahasa (1 village), Bolaang Mongondow (1 village), and Tomohon (3 villages, and 1 bushmeat market), and quantitative data collection was conducted in Minahasa (3 villages) and Bolaang Mongondow (1 village) districts only. The villages names have been coded as Village A (located in Bolaang Mongondow District), Village B, Village C, and Village D (all three in Minahasa District). Biological samples were collected from 254 participants who participated in the quantitative study (i.e. completed questionnaires) in Minahasa and Bolaang Mongondow from 2017 to 2018. As one of the main purposes of this study was to detect virus spillover from animals to humans, these two locations were selected so that human sampling could be conducted concurrently with wildlife sampling (i.e. 2 weeks before or after wildlife sampling and in a radius of 10 km) [26].

### Participants

The inclusion criteria for the quantitative study included adults (18 years of age and older) who provided informed consent and children (10–17 years of age) who agreed to provide assent with an accompanying parent or guardian who was able to provide informed consent. For the qualitative research, the interview participants included consenting adults living or working in the study area, with a 35–40% target enrollment for female participants. Children were not recruited for FGDs.

### Quantitative data collection

Informed consent was obtained from each participant before enrollment in the study. A set of questionnaires (S1 File) was administered to each enrolled participant to assess demographic conditions, work practices, and types of exposure to a range of animals: wildlife (bats, rodents, non human primates) and domesticated animals (swine, poultry, dogs) [27]. The questionnaires were in the Indonesian language, and local languages (Manado and Minahasan languages) were also used orally to make sure the participants fully understood the questions. The participants were randomly selected based on their availability at the time of data collection and willingness to be enrolled in the study.

### Qualitative data collection

The study team recruited a diverse sample of participants inclusive of different religions and cultural and socio-economic backgrounds. Purposive sampling was employed to identify participants who could discuss contact with domestic animals, wildlife, and bushmeat. The head of the villages were specifically asked to involve hunters or anyone who has contact with animals. Participation relied on their availability and willingness to be involved in this study. The semi-structured guides created for the interviews and FGDs were designed to be topically complimentary. The ethnographic interview instrument was structured to probe on 1) human movement, 2) socioeconomics, 3) biosecurity in the human environment, 4) illness, medical care/treatment and death of humans, and 5) human-animal contact. The FGD guide centered its questions on 1) contact and context, 2) illness in animals and humans, and 3) rules and restrictions around wildlife and waste management [27].

Interviews were conducted on a one-on-one basis in a quiet and private place in an area where there was no other individual present within a 10-ft distance, while FGDs were led by at least two team members who served as facilitators. Interviews and FGDs lasted between 60 and 90 minutes. The focus groups were stratified primarily by occupation, representing market vendors, hunters, and collectors. All qualitative data collection events were audio recorded. The audio files of the ethnographic interviews and FGDs were transcribed in the Indonesian language and then translated into English. All physical and digital materials were de-identified and securely stored.

### Biological sample collection, molecular viral screening, and serological detection

Biological samples were collected from 254 participants in Minahasa and Bolaang Mongondow districts during 2017–2018. The samples collected from each participant consisted of nasal swab, oropharyngeal swab, and blood. The swab samples were stored in Viral Transport Medium (VTM) and Trizol medium to preserved their quality and integrity. Five milliliters of blood were collected from each participant and stored in a vacutainer tube containing EDTA for further processing. The vacutainer tubes were then centrifuged at 3000 x g for 5 minutes to obtain serum. Total RNA, both from host and pathogen, if any, were extracted from the nasal swab, oropharyngeal swab, and serum blood samples of each participant using the Direct-Zol RNA Miniprep kits (Zymo research, USA). Procedures were performed according to the manufacturer’s instruction with elution volume of 60 μl. Prior to PCR, 4 μL of extracted RNA were converted to cDNA using GoScript Reverse Transcription System (Promega, USA). The cDNA was then used as a template for conventional PCR targeting five viral families including Coronaviridae, Filoviridae, Flaviviridae, Orthomyxoviridae and Paramyxoviridae as previously described [28]. All cDNAs from nasal, oropharyngeal and serum specimens were tested for all five viral families. These broadly reactive PCR assays were design to detect known and potentially novel viruses as described previously (Mawuntu 2018). Two μL of the cDNA produced were used as template for a total of 25 μL PCR reactions of Go Taq Green Polmerase Master Mix (Promega, USA). Synthetic DNA plasmid were used as positive controls. All PCR products were analyzed using electrophoresis in 1.5% agarose gel. Visualization of positive band was performed using Gel Imaging BioRad Gel Doc XR System and Quantity One 1-D Analysis Software (Bio-Rad, USA). Positive PCR products were purified and sequenced using BigDye Terminator v 3.1 (Applied Biosystems, USA) to confirm. Sequencing results were then analysed and compared with the BLAST database for sequence similarity.

Serological detection for SARS-CoV-2 antibodies was performed using the SARS-CoV-2 Surrogate Virus Neutralization Test (sVNT) kit (GenScript, Jiangsu, China). Prior to procedure, serum was heat-inactivated at 56 °C for 30 minutes. Procedures were conducted in accordance with manufacturer’s instructions. All samples were tested in duplicates. In brief, neutralization reaction were done by incubating controls and inactivated serum with horseradish peroxidase-conjugated RBD (HRP-RBD) at 37 °C for 30 minutes. The reactions were then added to capture plate coated with hACE2 and incubated at 37 °C for 30 minutes. Plates were washed to remove neutralizing antibody HRP-RBD complexes while unbound HRP-RBD and HRP-RBD bound to non-neutralizing antibodies were captured on the plate. 3,3′,5,5′-Tetramethylbenzidine (TMB) substrate was added followed by Stop Solution to quench the reaction. Inhibition percentages greater than or equal to 30% were considered as positive detection for neutralizing antibodies for SARS-CoV-2 and less than 30% as negative, following the manufacturer’s recommendations.

### Data analysis

Data from questionnaires were recorded manually and entered into EIDITH, the PREDICT study database system. R version 3.5.2 and several associated R packages (stats, dplyr, tidyverse, descr, eidith, arsenal, tadaatoolbox [29–34]) were used to clean and analyze the data. Descriptive analysis with Chi-square tests and Kruskal Wallis tests were used to summarize participant demographic data, while binomial logistic regressions were used to explore the relationship between health-seeking behaviors and potential influencing factors. Chi-square and Fisher’s exacts were used to analyze the statistical significance of parameters of interests. A *p*-value of less than 0.05 was considered as statistically significant. Missing values were omitted before the regression analysis process. We used Graphpad Prism version 9.2.0 for macOS to generate heatmap plot and bar graphs.

Trained behavioral risk researchers coded the interview and focus group transcripts using MAXQDA (Plus 12) qualitative coding and analysis software. Coding was completed using a codebook that thematically paralleled and expanded upon the topics in the quantitative questionnaires. Throughout the transcript coding process, the codebook was iteratively modified through the addition, removal, and modification of codes until saturation was achieved [35]. The data were then thematically analyzed [36].

## Results

### Participant and household characteristics

Forty-six semi-structured ethnographic interviews, which were conducted in Minahasa (5 interviews), South Minahasa (26 interviews), North Minahasa (3 interviews), Bolaang Mongondow (5 interviews), and Tomohon (7 interviews), included 24 (52%) females and 22 (48%) males with an overall mean age of 40 (sd = 11.7). Occupational representation was inclusive of 21 hunters (46%), 16 wildlife vendors (35%), 5 consumers (11%), 3 collectors (7%), and 1 transporter (2%). We conducted two FGDs with a total of 29 participants (16 vendors in the first FGD in Tomohon; 11 hunters and 2 collectors in the second FGD in South Minahasa). Hunters were predominantly male while vendors were predominantly female. The age ranges of the participants were 22–67 for ethnographic interviews and 18–50 years old for FGDs. Ethnographic interview participants’ level of education varied and included completion of primary school (10/46, 22%), secondary school (18/46, 39%), no formal education (5/46, 11%), and missing education data for 13 participants (28%). Almost all participants were married (91.3%; 42/46). We did not collect education and marital status data from FGD participants. Questionnaires were administered to 477 targeted participants for quantitative data collection (Table 1). The mean age of the participants were 48.9 (sd = 15.7) for Village A, 50.6 (sd = 16.8) for Village B, 39.0 (sd = 17.1) for Village C, and 49.1 (sd = 16.7) for Village D. The mean age for overall participants was 47.0 (sd = 17.0). The mean of overall crowding index was 1.3 (sd = 0.5) with range from 0.3 to 3.5. Age, primary livelihood, and crowding index, and presence of a dedicated location for waste differed significantly among the localities included in the quantitative study (Table 1).

**Table 1:** Demographic and household characteristics of the quantitative study participants

	Village A (*n* = 154)	Village B (*n* = 112)	Village C (*n* = 111)	Village D (*n* = 100)	Total (*N* = 477)	*p*-value Chi-square & Kruskal wallis
Age						**< 0.001**
under 24	10 (7%)	10 (9%)	27 (24%)	8 (8%)	55 (11%)	
25 to 54	102 (66%)	54 (48%)	68 (61%)	57 (57%)	281 (59%)	
over 55	42 (27%)	48 (43%)	16 (14%)	35 (35%)	141 (30%)	
Gender						0.057
Female	89 (58%)	66 (59%)	76 (68%)	72 (72%)	303 (64%)	
Male	65 (42%)	46 (41%)	35 (32%)	28 (28%)	174 (36%)	
Highest education						0.250
None + primary school	56 (36%)	34 (30%)	47 (42%)	32 (32%)	169 (35%)	
Secondary school + college/university/professional	98 (64%)	78 (70%)	64 (58%)	68 (68%)	308 (65%)	
Primary livelihood						**< 0.001**
Crop production	52 (34%)	14 (12%)	34 (31%)	47 (47%)	147 (31%)	
Domestic animal related business	4 (3%)	2 (2%)	5 (5%)	1 (1%)	12 (3%)	
Homemaker	32 (21%)	40 (36%)	42 (38%)	28 (28%)	142 (30%)	
Non-animal related business	44 (29%)	42 (38%)	11 (10%)	17 (17%)	114 (24%)	
Unemployed/student/child	6 (4%)	12 (11%)	19 (17%)	7 (7%)	44 (9%)	
Wildlife related business	16 (10%)	2 (2%)	0 (0%)	0 (0.0%)	18 (4%)	
Crowding index						**< 0.001**
Mean (SD)	1.5 (0.6)	1.3 (0.5)	1.3 (0.5)	1.1 (0.5)	1.3 (0.5)	
Range	0.4–3.5	0.33–2.33	0.5–3.0	0.3–2.7	0.3–3.5	
Dedicated location for waste						**< 0.001**
No	40 (26%)	0 (0%)	7 (6%)	3 (3%)	50 (10%)	
Yes	114 (74%)	112 (100%)	104 (94%)	97 (97%)	427 (90%)	

### Diversity in animal contact

**Fig. 2:**
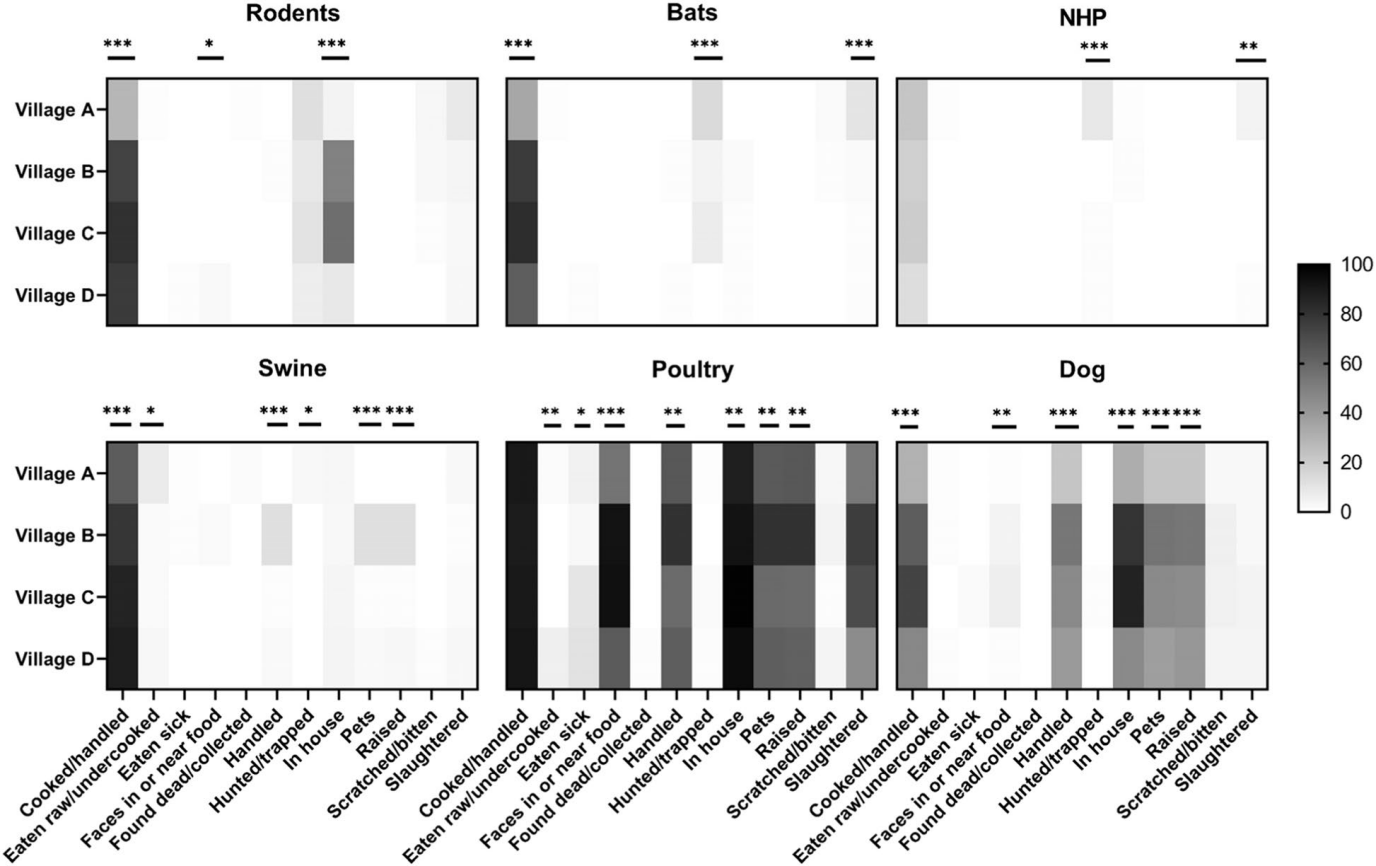
Type of contact with wildlife and livestock of participants (*N* = 477) from four localities where quantitative data collection was conducted. Respondents could choose more than one type of contact for each taxa. *0.01 < *p*-value < 0.05, ** 0.001 < *p*-value< 0.01, ***p-value < 0.00

Almost all participants from the quantitative data collection had contact with animals within the previous one-year time period. Participants reported coming into contact with animals through various activities. Overall, the most reported contact was with poultry and swine, both common domestic animals in the areas. A total of 68% (362/477) of participants reported having had contact with wildlife (bats, rodents, and/or primates), mostly through cooking/handling (Fig. 2/S2 File. Table S1). Participants from Village A in Bolaang Mongondow district reported having significantly more contact with bats and non human primates through hunting/traping or slaughtering activities compared to other villages (Chi-square test, *p* <  0.05).

As a result of the sampling design for the qualitative data collection, contact with some form of wildlife or bushmeat was represented in all of the interviews. From handling animals in hunting territories to coming into contact with live and dead wildlife in local markets, respondents detailed the many ways in which proximity to and direct contact with various taxa was a part of their daily lives.

With regard to wild animals in the context of their work responsibilities as hunters and wildlife vendors, reports of taxa contact included rats (commonly, white-tailed rats) (37/46, 80%), bats (29/46, 63%), wild boar (30/46, 65%), primates or “yaki” (Celebes crested macaques) (13/46, 28%), cuscuses (8/46, 17%), snakes (16/46, 35%), and anoas (local wild cows) (5/46, 11%). Across the majority of the interviews, it was more common for hunters and vendors to work with multiple wildlife taxa rather than just one.

Respondents were probed on local meat consumption practices and preferences regarding wildlife. A joking sentiment shared among multiple participants was that they ate everything that came from the forest, or everything that they could hunt or sell. In the handful of instances where respondents described an aversion to specific taxa, snakes and monkey were most commonly identified.

*Respondent 1: “I cannot eat roromeha [black snake with red lines on the belly/cobra].”*

*Respondent 2: “I avoid eating monkey yaki.”*

*Interviewer : “Why?”*

*Respondent 2: “Sometimes I feel pity for the monkey yaki.”*

*Respondent 1: “There is poison sometimes in the snake.”*

- Focus Group Discussion with vendors.

Most respondents described having pets or other animals around their home for protection, rearing, and companionship. Many of the respondents reported currently having a dog or multiple dogs around their dwellings, and many also reared chickens. There were also observations shared regarding rare and exotic pet ownership. These pets, typically various species of birds, were considered expensive animals and were thought to have been brought over from nearby islands or other locations.

### Knowledge

#### Zoonotic transmission

The interviews and FGDs also explored respondent knowledge about disease transmission from animals to humans. When probed about any diseases that they understood to be of animal origin, many respondents identified avian influenza as one that they were familiar with, typically alongside rabies. Those who made this link frequently associated avian influenza explicitly with birds and chickens.

*Interviewer: “Do you know anyone who got sick from animals?”*

*Respondent: “Yes, rabies when bitten by dogs.”*

*Interviewer: “What else?”*

*Respondent: “Bird flu from chicken or any birds.”*

- 48-year-old male wildlife vendor.

However, very few respondents were able to speak in detail about animal to human transmitted diseases other than rabies and bird flu. While respondents did often nominally reference diseases such as SARS, MERS, and Zika, this appeared to be driven by media exposure. Of note, respondents identified television, news, social media, and word-of-mouth as the means through which they received health messages about diseases.

*Interviewer: “Do you know anyone who got sick from animals?”*

*Respondent: “Yes, rabies, SARS, MERS, and the last is Zika virus…I always read the news on facebook and other social media that they circulate in the group.”*

- 32-year-old female wildlife consumer.

We also explored the respondent’s ability to identify sick animals. The respondents were mostly aware of sicknesses in dogs or poultry and reported to know the symptoms that indicated poor health.

*Interviewer: “How do you know when your dog or chicken get sick?”*

*Respondent: “Normally they look not healthy, the dog tail going below the stomach, or chicken getting spots in the head and runny nose.”*

- 36-year-old female wildlife vendor.

To improve our understanding about respondents’ experiences related to zoonotic events, we asked the FGD group participants about any memorable event related to animal and sickness they or their relatives or friends had experienced.

*“… in two weeks the puppy suddenly died and they buried the puppy in their front yard. The next day there was news that there was a case of a woman who died because of rabies from the dog. So, they were afraid with the puppy that just died the day before because it was a very sudden death. They dug the grave and brought the puppy head to the laboratory in [redacted locality]. After a day, the results came that the dog was positive for rabies. So, the recommendation from the lab was that everyone who had physical contact and got scratched and bitten by the puppy should receive anti-rabies injections…” (Focus Group Discussion 1).*

#### Perception of the cause of sickness

In the survey, we also asked the respondents about their perception of the cause of sickness. Only 1% (6/477) of participants said that contact with wild animals can cause sickness, 24% of (114/477) participants said that they do not know the cause of sickness, and for more than half of the respondents (61%), perceptions on the cause of sickness varied, including fatigue, change of weather, pollution, and pathogens (S2 File. Table S2).

### Attitude

#### Risk of zoonotic transmission

As part of quantitative data collection, we asked respondents if there was a risk associated with slaughtering or butchering wild animals while having an open wound. Among those who responded affirmatively, we then asked what the specific risk was when slaughtering or butchering with an open wound. There was a significant difference between participants across the study sites in answering the question (Chi-square test, *p* <  0.001). More than a half of the partipants in Village A (57%, 88/154) and a third of the participants in Village D (39%, 39/100) reported that there is no risk associated with slaughtering/butchering activities while having open wound. These findings were in contrast with what was reported in Village B and Village C, where only a minority of the participants (4%, 5/112 and 5%, 6/111, respectively) reported that there is no risk associated with slaughtering/butchering activities while having open wound. Overall, among participants who answered “yes”, the majority of them did not know what the risks were and only 2% (11/477) of participants had the perception that it could infect them with a disease and and 7% (34/477) believed it could make them sick (Table 2). The presence of a dedicated location for waste which reflected the participants’ attitude related to risk of zoonotic transmission differed significantly between the villages (*p* <  0.001), with a quarter of participants from village A reporting that they did not have a dedicated location for waste (Table 1). When asked if they were worried about disease outbreaks in live animals in their local market, 90% (430/477) of the participants in all villages said they were worried (Table 2).

**Table 2:** Attitude or beliefs on zoonotic disease risk among study participants

Characteristics	Village A(*n* = 154)	Village B(*n* = 112)	Village C(*n* = 111)	Village D(*n* = 100)	Total(*n* = 477)	*p*-value Chi-square test
Knows of Risks Associated with Open Wound						**< 0.001**
No	88 (57%)	5 (4%)	6 (5%)	39 (39%)	138 (29%)	
Yes^a^	66 (43%)	107 (95%)	105 (95%)	61 (61%)	339 (71%)	**< 0.001**
There are risks, but do not know what they are	58 (38%)	98 (87%)	97 (87%)	51 (51%)	304 (64%)	
It can infect you with a disease	0 (0.0%)	3 (3%)	5 (5%)	3 (3%)	11 (2%)	
It can make you sick	9 (6%)	10 (9%)	6 (5%)	9 (9%)	34 (7%)	
It can poison you	0 (0%)	0 (0%)	2 (2%)	0 (0%)	2 (0.4%)	
Worried About Disease in Animals at Market						**< 0.001**
No	26 (17%)	3 (3%)	3 (3%)	15 (15%)	47 (10%)	
Yes	128 (83%)	109 (97%)	108 (97%)	85 (85%)	430 (90%)	

^a^Participants could choose more than one answer. ^#^ The *p*-values were calculated with Chi-Square tests. The significant *p* values are in bold (*p* < 0.05)

In addition, we ran a logistic regression analysis to understand which socio-economic factors (gender, age, education, study site, and livelihood) affected the participants’ beliefs on the risk of zoonotic disease transmission (Table 3). The analysis confirmed that the study sites have an effect on the participants’ perception about the risk of working with open wounds and of disease outbreaks in animal markets, with participants from Village A and those whom reported themselves as homemakers being less aware of the risk. Participants from Village B and Village C were also more aware about the risk of disease in animals in markets (*p* <  0.001). Furthermore, male participants, homemakers, and those whose primary livelihood was in non animal related business were less likely to worry about disease outbreaks compared to female participants. Level of education, age, crowding index, and the presence of dedicated location for waste did not affect participants’ attitude or beliefs around zoonotic disease risk.

**Table 3:** Logistic regression analysis to understand the association between socio-economic factors and participants’ beliefs about the risk of zoonotic disease transmission

Gender	Knows of Risks Associated with Open Wound(*n* = 477)^a^Adj-R2 = 0.293			Worried About Disease in Animals at Market(*n* = 477)^a^Adj-R2 = 0.078		
	OR	95% CI	*p* value	OR	95% CI	*p* value
Female	Ref					
Male	0.86	0.47–1.57	0.625	**0.24**	**0.09–0.56**	**0.001**
**Age**						
Under 24	Ref					
25 to 54	0.99	0.38–2.48	0.99	1.64	0.46–5.09	0.42
over 55	0.88	0.32–2.38	0.80	2.02	0.51–7.24	0.29
**Highest education**						
None + primary school	Ref					
Secondary school + College/university	1.14	0.64–2.01	0.66	1.65	0.78–3.46	0.18
**Study sites**						
Village A	Ref					
Village B	**34.47**	**13.51–107.84**	**< 0.001**	**9.47**	**2.95–42.61**	**< 0.001**
Village C	**30.47**	**12.31–88.71**	**< 0.001**	**8.58**	**2.59–39.66**	**0.001**
Village D	**2.01**	**1.11–3.69**	**0.02**	0.93	0.41–2.10	0.86
**Primary livelihood**						
Crop production	Ref					
Domestic animal related business	4.17	0.59–84.79	0.21	0.57	0.08–11.86	0.63
Homemaker	**0.31**	**0.15–0.64**	**0.002**	**0.19**	**0.05–0.55**	**0.003**
Non-animal related business	1.09	0.55–2.16	0.81	**0.35**	**0.13–0.89**	**0.02**
Unemployed/student/child	0.54	0.18–1.66	0.27	0.68	0.16–3.59	0.62
Wildlife related business	0.69	0.21–2.11	0.52	0.49	0.13–2.09	0.31
**Crowding index**	0.93	0.58–1.51	0.78	1.32	0.71–2.56	0.39
**Dedicated location for waste**						
No	Ref					
Yes	1.93	0.94–4.07	0.07	1.32	0.49–3.23	0.56

^a^Missing values, if any, were omitted before the regression analysis process

### Practice

#### Health-seeking behavior

In the survey, we asked the participants about what they did the last time they got scratched, bitten, or cut while butchering or slaughtering an animal. Forty five percent (219/477) of participants answered that they treated the wound either by visiting a doctor or cleaning it with soap. We performed a logistic regression analysis to better understand socio economic factors associated with treatment behavior among participants (S2 File. Table S3). The education level of mothers contributed to the treatment behavior, with higher maternal education being associated with a greater likelihood that the participant would treat their wounds (OR = 1.75, *p* value = 0.04). Furthermore, participants from Village B, C, and D were more likely to treat wounds received from butchering or slaughtering activities compared to those from Village A (OR = 3.22, 3.56, and 1.89; *p* value = < 0.001, 0.001, 0.05 respectively).

**Fig. 3:**
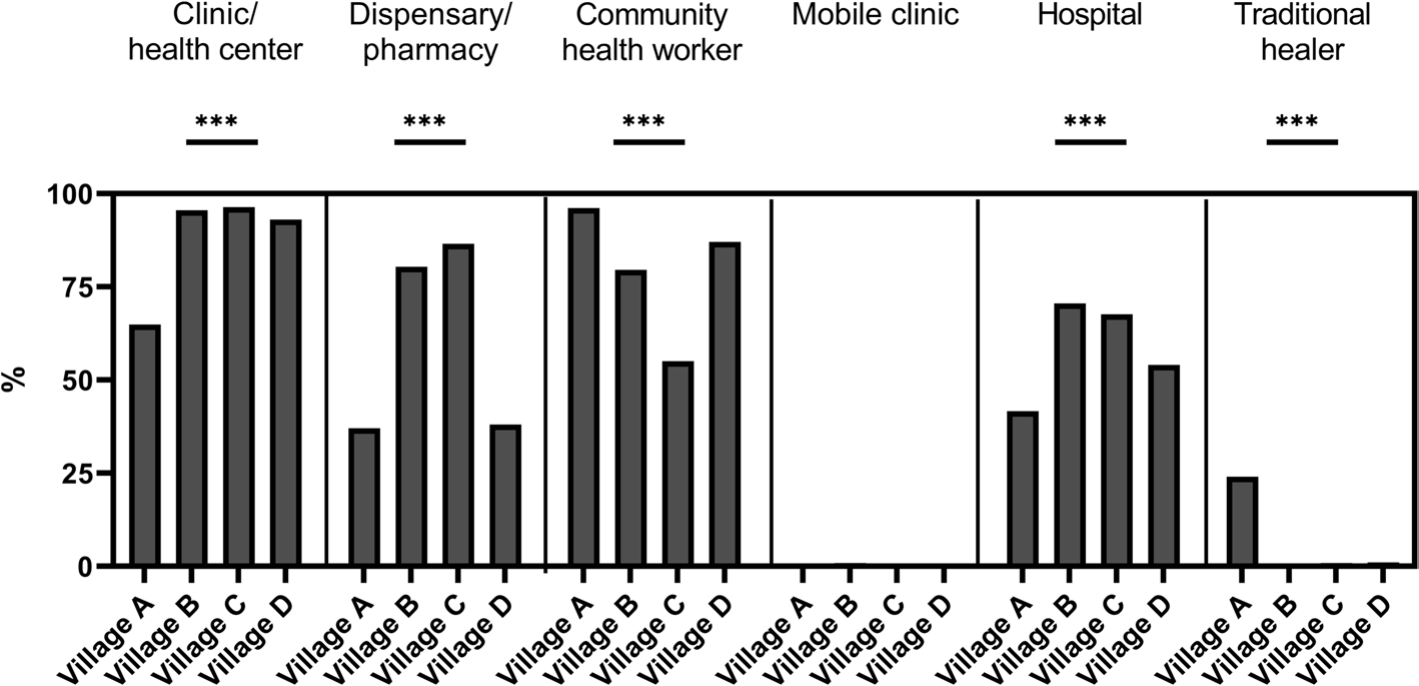
Treatment seeking behaviour of the study participants. *0.01 < *p*-value < 0.05, ** 0.001 < *p*-value< 0.01, ***p-value < 0.001

Overall, survey participants reported seeking treatment from the following institutions and actors for medical conditions they have experienced: clinics/health centers (85%, 407/477), community health workers (81%, 385/477), hospitals (57%, 272/477), traditional healers (8%, 39/477), and dispensaries or pharmacies (59%, 281/477) (Fig. 3/S2 File. Table S4). However, we observed variations among sites. Participants from Village A were less likely to visit the clinic/health center or hospital compared to other villages (*p* <  0.001) while 24% (37/154) of these participants reported seeking treatment from a traditional healer, contrasting with other villages where very few or no participants chose this option (*p* <  0.001).

**Fig. 4:**
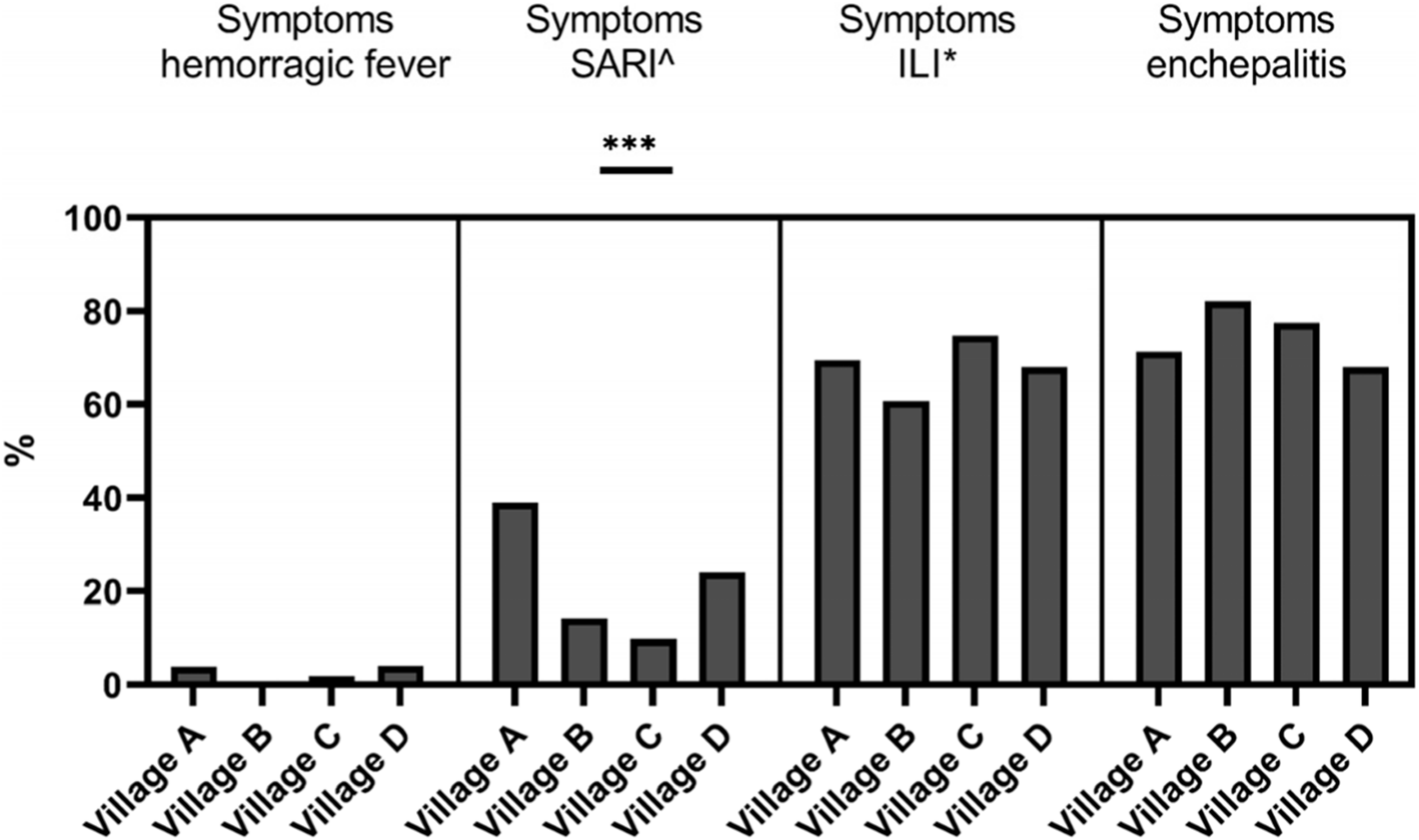
Self-reported unusual symptoms in the past year period. ^ SARI = Severe Acute Respiratory Infection, * ILI = Influenza like illness. *0.01 < *p*-value < 0.05, ** 0.001 < *p*-value< 0.01, ***p-value < 0.001

Participants were also asked to report unusual illness symptoms they experienced in the past year before the questionnaire interview. The most reported symptoms from participants in all villages were those associated with Influenza like illnesses (ILI) (68%) and enchepalitis (75%) (Fig. 4/S2 File. Table S5). Participants from Village A reported more Severe Acute Respiratory Infection (SARI) symptoms than other villages (*p* <  0.001).

When testing the correlation between types of contact with wildlife and self-reported symptoms, we observed that hemorrhagic fever symptoms were associated with wildlife hunting/trapping (*p* = 0.01) and being scratched or bitten by wild animals (*p* = 0.0007) (S2 File. Table S6). SARI and ILI symptoms were both significantly associated with wildlife hunting/trapping (*p* = 0.01) and wildlife slaughtering (*p* = 0.001) for SARI and wildlife slaughtering (*p* = 0.0165) and wildlife cooking/handling (*p* = 0.0381) for ILI (S2 File. Table S6).

In the qualitative data, an integral component of understanding risk perception involved probing on wildlife bites and scratches, and subsequent health-seeking behaviors. Respondents were generally open to discussing the types of animals they had been bitten or scratched by, and how they responded to their wounds. Since this sample of respondents was purposively recruited from the wildlife markets and value chains, nearly everyone reporting bites was not unexpected. Reports of rat and dog bites were most common among this group, followed by bat bites, and in some cases, bites by snakes and wild boars. Rat bites were especially described as bloody and painful.

*“…, I used to be bitten by rats. I just had fever which is what normally happened when got bitten by the rats or bats. I did not visit doctor, only used the leaves and grass available in the field and forest.”*

- 45-year-old male hunter.

Respondents also described instances of getting scratched while handling rats and bats, in addition to dead animal parts such as bones and claws. Respondents described wounds from live animals, but also from processing dead or frozen wildlife. Among those who reported being scratched by wildlife and bushmeat (all of whom were vendors) either directly or indirectly, bats accounted for the most injuries.

*“…I did not pay attention when separating the frozen bats, the nail was coming out from the bag and hurt me…I just put leaves that we usually put on the wound.”*

- 22-year-old female wildlife vendor.

As these excerpts demonstrate, it was uncommon for respondents to seek clinical treatment for animal bites and scratches. While seeking clinical care appeared to be a final solution for most respondents, especially for more urgent health complications, many indicated that they also relied upon traditional methods/natural remedies, self-medication, and seeking care first from people such as midwives and “mantri” (senior nurse). Reasons for why certain health-seeking decisions were made also varied, including preference, perceived injury severity, matters of convenience (e.g. traditional medicines take more time to seek out), and cost.

*“I was sick because of the frozen bats. I like to separate the bats quickly, so I use a kind of wooden hammer. When I have to get the bats separately, I did not know if I got punctured on my hand by the nail of the bats. After I finished, when I washed my hand, I saw the wound and the blood inside the wound. After three days, I got high fever and my hand swollen because of the infection, my hand was very big and I could not do my job anymore. So, I went to the doctor and I couldn’t work for two weeks.”*

- Focus Group Discussion with Vendors.

#### Hygiene practices

We assessed participant hygiene practices through handwashing and found that while some wash their hands before eating or any time when they feel dirty, others reported that they did not wash their hands because of the lack of water or time.

*“No, I don’t. There is no water sometimes I just use a spoon [in a forest context].”*

- 52-year-old male hunter.

Many of the participants broadly described disposing of slaughtered animals, viscera, and dead animals found in the forest into natural bodies of water. With regard to the disposal of dead animal bodies and body parts, there were also descriptions of animal leftovers, including that of wild animals, being fed to domestic animals, or of viscera being eaten by animals in the marketplace or near slaughter locations.

*Respondent: “Yes, the water flows from my table drink by the dogs or chickens”*

*Respondent: “Yes, the birds drink the bloody water…”*

- Focus Group Discussion with Vendors.

#### Animal vaccination practices

Nearly all respondents in the study were able to describe witnessing or hearing about some kind of animal outbreak or die-off in their communities and markets. The majority of outbreaks were attributed to chickens, with some respondents remarking that chicken deaths were an approximately yearly occurrence. There were references to municipal support for outbreaks, with stories indicating vaccination interventions and reporting. Vaccinations were recognized as a way to actively protect chickens against the regular die-offs. However, a few respondents also described actively not reporting their chicken die-offs to local authorities, and while there was a general awareness of the existence of vaccination programs for chickens and dogs provided by the government, there was not a clear corresponding uptake of the vaccination programs. Some participants reported that they vaccinated their pets or poultry regularly to avoid animal death during outbreaks, while others refused to vaccinate due to economic reasons. Participants also described various ways of administering vaccines, including self-administration, through veterinary visits, or by the government officials.

### Molecular viral screening and serological detection

Out of the 912 samples from 254 participants tested for five viral families, only one oropharyngeal swab sample was tested positive for paramyxovirus. We further sequenced the positive sample, and it was identified as Measles virus. The positive case was from an older female who works in crop production living in Village D. Additionally, we found one serum sample positive for SARS-CoV-2 neutralizing antibody test with an inhibition percentage of 40% from a participant living in the same village. The sample was obtained in May 2018 from an adult female participant who worked at a non-wildlife restaurant business.

## Discussion

Our study provides important baseline information on the variability in knowledge, attitudes, and behavior regarding zoonotic disease risk in communities in North Sulawesi Province, Indonesia, collected prior to the emergence and spread of COVID-19. North Sulawesi is famous for its culinary culture in consuming bushmeat, but few studies have used quantitative or qualitative behavioral research methods to quantify this [12, 13]. Rats, bats, non-human primates, snakes, anoas, and wild boar were the most common wild animal taxa handled by the study participants, in line with their role as hunters, vendors, or consumers in the bushmeat trade market and value chain. The preference for certain animal species has not changed in the past decades, as reported by previous publications [37]. In addition to those wildlife taxa, livestock such as poultry and swine were also often mentioned during the questionnaire administration. These results are not surprising given that the locations of this study were in rural areas, so it is very common for people to have livestock around their homes. The results from this study demonstrated that bushmeat hunting is a male-dominated activity. This finding is similar to a report from Cameroon, where males conduct a majority of hunting activities compared to women [27]. Male hunters were therefore among the highest risk group for exposure to zoonotic diseases (especially labile RNA viruses) because of close contact with the live animals who could be actively shedding virus [23]. However, for pathogens that may be transmitted after an animal is deceased (e.g. more stable DNA viruses), women who make up a majority of butchers and wildlife vendors may be at greater risk. Viral screening of samples collected from 254 participants in two districts using a conventional PCR approach did not detect any zoonotic virus spillover in communities targeted in this study, as only one sample was positive for measles.

Serological testing for SARS-CoV-2 revealed one positive sample. As SARS-CoV-2 had not emerged yet at the time of sample collection (May 2018), this could correspond to a cross-reaction with another SARS-related coronavirus (sarbecovirus). It has been demonstrated that the SARS-CoV-2 sVNT assay we used can differentiate antibody responses to SARS-CoV-2 from other human CoV infections such as 229E/NL63, MERS, and OC43 [38]. The only detected cross-reactivity was with recently collected sera from patients who were infected by SARS-CoV in 2003 [38]. This is not surprising given the close genetic relatedness of these two sarbecoviruses. We cannot rule out that the study participant with positive serological results had a history of SARS-CoV infection. Indonesia reported two probable and seven suspected cases during the period of 1 March to 9 July 2003, all of which were imported cases with reported travel history from Singapore and China [39]. This finding is supported by a previous study that found one individual sample in rural China to be positive for exposure to sarbecoviruses in 2018 prior to COVID-19 and in a population not exposed to SARS-CoV [40], and supports recent modeling work that suggests ~ 50,000 people per year could be infected with SARS-realted viruses from direct bat-human contact [9]. Blood and oropharyngeal swab samples from this participant were tested for coronaviruses using two molecular (degenerate PCR) tests [41, 42] but both samples were negative. A study that used the sVNT assay also reported 50% cross-reactivity with syphilis. Serology testing validation or cross checking need to be performed to confirm the positive result [43].

In regards to knowledge of zoonotic disease transmission, rabies and avian influenza were mentioned by the majority of the participants because they experienced it firsthand or it occurred in their neighborhoods. The participants were able to describe these disease symptoms with ease, and generally understood the necessary steps that needed to be taken if these occurred. This is similar to another study which mentioned that past experience of a disease outbreak increased awareness and knowledge of the people in the area [44]. In addition, the participants were aware of vaccination programs for chickens and dogs regularly run by government officials. Prevention strategies are critical considering the huge losses caused by the bird flu in the past and the fatality rate of rabies which is close to 100% [11, 45].

Perception of wild animals as the cause of sickness was very low in the studied communities. This finding is concerning as such knowledge can be indicative of greater community awareness around zoonotic transmission. More than half of the participants had protective beliefs about the risk associated with slaughtering or butchering while they have an open wound, but they lacked knowledge about what the specific risks were. This partial knowledge highlights potential entrypoints for educational interventions, particularly among those specifically involved in the wildlife value chain. The majority of participants reported that they had concern about disease outbreaks in live animal markets, in contrast with a study in Tanzania where participants were skeptical about the reality of zoonotic transmission [46].

Our results showed that although participants were aware of the importance of visiting a doctor when sick and experiencing symptoms (e.g. fever) after contact with or getting wounded by wild animals, some of them preferred alternative medicine based on medicinal plants instead of professional care, at least for initial treatment. These findings are similar to the study conducted in southeast Nigeria among bushmeat hunters and traders [47]. From the logistic regression analysis, we identified various factors that associated with treatment behaviors among participants. However, significant differences in the attitude or beliefs around zoonotic disease risk and in health seeking behaviors were observed across our study sites in North Sulawesi. Participants from Village A in Boolang Mongondow district were both significantly less aware of the risk associated with wildlife slaughtering/butchering activities and less likely to visit a health center or a hospital to seek treatment. This is particulary alarming as this locality was characterized by significantly higher contact with bats and non human primates through hunting/trapping or slaughtering activities, a higher crowding index, and a higher proportion of houses without dedicated location for waste than the other study sites included in our quantitative study, indicating that the risk of zoonotic spillover is high in these communities. Cultural and socio-economic differences may be related to this heterogeneity in terms of perception of zoonotic risk in North Sulawesi. Old belief systems, animism, and traditional medicine use are strong in Bolaang Mongondow district while Minahasan localities are more open to change and modernism as reported by a previous study [48]. This district is also characterized by lower human development index (HDI) when compared to Minahasa district. The HDI is based on three components consisting of life expectancy, education, and per capita income. According to the North Sulawesi Bureau of Statistics (BPS Sulawesi Utara), the 2018 HDI of Bolaang Mongondow district was 66.91 while the HDI of Minahasa district and North Sulawesi province as a whole from the same periode were 74.97 and 72.20 respectively [49].

Some other risk behaviors in relation to hygiene and vaccination practices were reported by participants, such as dead animal disposal in water (river or swamps), which can cause water contamination and lead to disease transmission [50]. Moreover, hand hygiene awareness after touching animals needs to be improved, along with the limited facilities and infrastructure for clean water and handwashing [27]. Proper hand washing practices have been reported in several studies as effective to prevent disease transmission such diarrhea and seasonal influenza [51, 52]. Vaccination practices varied among the participants. Some of them vaccinated their livestock/pets regularly while a few of them reported not giving vaccinations for economic reasons. These findings indicate that information on the importance of vaccination needs to be improved in the community.

There are several limitations in our study: due to the purposive sampling design of the interviews and FGDs, the qualitative findings should not be interpreted as representative of broader behavioral risk patterns across the studied regions or Indonesia as a whole. It is also important to note that FGDs were not conducted in all districts where interviews were obtained. Additionally, as the hunting and sale of certain types of wild animals are locally prohibited, it is possible that respondents may have held back details of their interactions with these taxa.

## Conclusions

Our study gives an understanding on the variability of knowledge, attitudes, and practices related to zoonotic disease risk among wildlife trade communities in North Sulawesi. Findings from this study could be used by the authorities to further develop intervention strategies and policy recommendations related to the mitigation of viral zoonotic disease emergence and transmission. Activities such as the integration of zoonotic disease risk education into primary and secondary education curricula from health agencies need to be intensified and conducted on a regular basis to improve the awareness of zoonotic risk transmission in high-risk communities in North Sulawesi Province. Communities operating at high-risk interfaces with lower perceptions of zoonotic disease risk transmission may be populations in which sensitively adapted education interventions can have great public health impacts. In addition, social media can be used as a tool for health promotion related to zoonotic diseases risk prevention. Other strategies based on alternative economic resources also need to be considered to limit the interaction between animals and humans, and will directly or indirectly affect conservation goals [16]. We detected evidence of potential SARS-related zoonotic virus transmission in a single individual sampled in 2018. Prospective cohort studies to follow the course of suspected zoonotic disease among high risk groups (e.g. wildlife hunters) through syndromic and laboratory based surveillance in collaboration with local health facilities and authorities need to be considered to rapidly identify future spillover events when they occur.

## Supplementary Information

**Additional file 1: S1 File.** Questionnaires - Quantitative data collection.

**Additional file 2: S2 File. Table S1.** Type of contact with wildlife and livestock of participants for quantitative data collection (*n* = 477). **Table S2.** Perception of the cause of sickness. **Table S3.** Logistic regression analysis on potential social economic factors contributed to wound treatment behavior. **Table S4.** Treatment seeking behavior of the study participants. **Table S5.** Self-reported unusual symptoms in the past year period. **Table S6.** Correlation between wildlife contacts and self-reported symptoms.

## Data Availability

The dataset used and/or analysed during the current study are available from the corresponding author on reasonable request.
