# Supplementary material for: Knowledge, attitudes, and practices associated with zoonotic disease transmission risk in North Sulawesi, Indonesia
Source: One Health Outlook. 2022 Jun 3;4:11. doi: 10.1186/s42522-022-00067-w (PMC9162794; doi:10.1186/s42522-022-00067-w)
Supplement: Supplementary file 1 — Additional file 1: S1 File. Questionnaires - Quantitative data collection. [file 42522_2022_67_MOESM1_ESM.pdf]

Directions for selecting modules for the interview

Select module(s) identified in Question 2 (if any).

Conduct the following QUICK CHECKS.

1. Add the primary work activity module (Question 25)

2. Add the Temporary Settlement Module if Temporary Settlement is NOT selected in Question 2 and dwellings is NOT permanent (Question 15)

3. Add Hunter Module if hunter/trapper/fisher is selected in Question 25 or “yes” to Question 55.

Livelihood Module Table (based on response to Question 25)
Complete the module that corresponds with the livelihood chosen as follows:

extraction of minerals, gas, oil, timber – extractive industry module
crop production – crop production module
wildlife restaurant business – wildlife restaurant module
wild/exotic animal trade business – market and value chain module
rancher/farmer animal production business - animal production module
meat processing, slaughterhouse, abattoir - animal production module
zoo/sanctuary animal health care – zoos & sanctuaries module
hunter/trapper/fisher – hunter module
nurse, doctor, traditional healer, community health worker - hospital or clinic health professional module

If no additional modules are selected, the interview is complete.

Human Questionnaire Form ID Instructions

Enter the Site and Event Form ID barcode number to the grid located at the top of page 1 of the Human Questionnaire Form.

Enter the Human Questionnaire Form ID barcode number to grid located at the top of each associated module.

The barcode is located at the bottom right hand corner of each page of the Human Questionnaire section and the Site and Event Characterization Form.

Use the number after the dash (-) and fill the grid with the numbers from top to bottom.

Human Questionnaire Form

EIDITH
V 1.2

Add Site and Event Form ID:

Site name and date:

____

(For reference only)

0	1	2	3	4	5	6	7	8	9
0	1	2	3	4	5	6	7	8	9
0	1	2	3	4	5	6	7	8	9
0	1	2	3	4	5	6	7	8	9
0	1	2	3	4	5	6	7	8	9
0	1	2	3	4	5	6	7	8	9

1. Consent Form Administered & Signed
   - ○ yes
   - ○ no

   Participant ID: ______________________

2. Description of Interview Location - Select all that apply.
   (To be completed by interviewer prior to administrative questionnaire.
   Prepare and download modules in advance.)
   - ○ Animal Production or Abattoir Site
   - ○ Crop Production Site
   - ○ Extractive Industry Site
   - ○ Market or Value Chain Site
   - ○ Temporary Settlement Site
   - ○ Tourism Site
   - ○ Wildlife Restaurant
   - ○ Zoos or Sanctuaries
   - ○ Hospital or Clinic - Health Professional
   - ○ Hospital or Clinic - Patient
   - ○ Natural Areas (eg. forest, urban park/garden)
   - ○ Other: ______________________

3. Date of interview ______________________

4. Begin time of interview ______________________
   (Example: 17:50)

5. End time of interview ______________________
   (Example: 19:20)

6. Where are you conducting this interview?

   Village/Town/City ______________ District ______________ Province/State ______________

   Latitude ______________________ Longitude ______________________

   Interviewer: Please collect GPS coordinates if administering using paper and pen.

7. Interviewer Observed Gender
   - ○ male
   - ○ female
   - ○ other

INTERVIEW/QUESTIONNAIRE BEGINS

Demographics Section (include observation question 7)

8. How old are you? ______________________
   If the exact age is unknown, enter the respondent's estimated age.

9. Where do you live?

   Village/Town/City ______________ District ______________ Province/State ______________

   Latitude ______________________ Longitude ______________________

   Interviewer: Probe for landmarks or nearest known site if area unknown.
   GPS coordinates to be identified and entered after completion of interview.

153893-108604

Human Questionnaire Form

10. How long have you lived there?
    Select one option.
    - ○ <1 month
    - ○ 1 month - 1 year
    - ○ >1 - 5 years
    - ○ >5 - 10 years
    - ○ >10 years

11. How many other people live in the dwelling where you live? ________
    Skip to question 14 if answer is 0.

12. How many in the dwelling are children less than 5 years old? ________

13. How many in the dwelling are male? ________

14. How many rooms are there in the dwelling where you live? ________
    (Do not include bathroom or kitchen)

15. Is the dwelling a permanent structure (that cannot be moved)?
    Interviewer: If answer is no, complete temporary settlement questionnaire.
    - ○ yes
    - ○ no

16. Do you get water from:
    Select all that apply.
    - ○ piped in water/water taps
    - ○ covered well
    - ○ uncovered well/pond/river
    - ○ water truck/rainwater harvest
    - ○ other: ____________________

17. Do you treat your drinking water?
    - ○ yes
    - ○ no

18. If yes, how do you treat your water?
    Select all that apply.
    - ○ boil
    - ○ filter
    - ○ add chlorine or bleach
    - ○ solar disinfection
    - ○ other: ____________________

19. Is your source for drinking water ever used by animals?
    - ○ yes
    - ○ no

20. In your dwelling is there a dedicated location for human solid waste/excreta?
    (Example: toilet, latrine, designated area)
    - ○ yes
    - ○ no

21. Do you have containers for storing food for the household?
    Select all that apply.
    - ○ yes, with covers
    - ○ yes, without covers
    - ○ no

153894-108604

Participant ID ________________
(For reference only)

Livelihood Section

In this section, I'd like to ask you about education and the kinds of work activities that you have done since this time last year.

22. What is the highest level of education you have completed?
Select one option. (Skip for Cameroon.)
   - ○ primary school
   - ○ secondary school
   - ○ college/university/professional
   - ○ none

23. What is the highest level of education that your mother completed?
Select one option. (Skip for Cameroon.)
   - ○ primary school
   - ○ secondary school
   - ○ college/university/professional
   - ○ none

24. Since this time last year what are the activities you have done to earn your livelihood?
Select all that apply.
   - ○ extraction of minerals, gas, oil, timber
   - ○ crop production
   - ○ wildlife restaurant business
   - ○ wild/exotic animal trade/market business
   - ○ rancher/farmer animal production business
   - ○ meat processing, slaughterhouse, abattoir
   - ○ zoo/sanctuary animal health care
   - ○ protected area worker
   - ○ hunter/trapper/fisher
   - ○ forager/gatherer/non-timber forest product collector
   - ○ migrant laborer
   - ○ nurse, doctor, traditional healer, community health worker
   - ○ construction
   - ○ other: ______________________

25. If more than one activity was selected, what is the activity on which you spent the most time since this time last year?*
Select one option.
   - ○ extraction of minerals, gas, oil, timber
   - ○ crop production
   - ○ wildlife restaurant business
   - ○ wild/exotic animal trade/market business
   - ○ rancher/farmer animal production business
   - ○ meat processing, slaughterhouse, abattoir
   - ○ zoo/sanctuary animal health care
   - ○ protected area worker
   - ○ hunter/trapper/fisher
   - ○ forager/gatherer/non-timber forest product collector
   - ○ migrant laborer
   - ○ nurse, doctor, traditional healer, community health worker
   - ○ construction
   - ○ other: ______________________

26. Which best describes your job position?
Select one option.
   - ○ manager/owner/foreman
   - ○ worker
   - ○ live and work at home independently (If chosen, skip to question 28)
   - ○ professional
   - ○ other: ________________

27. Where do you work?

Village/Town/City ____________ District ____________ Province/State ____________

Latitude ________________ Longitude ________________

Interviewer: Probe for landmarks or nearest known site if area unknown.
GPS coordinates to be identified and entered after completion of interview.

153895-108604

Participant ID ____________
(For reference only)

Medical History Section

In this section, I'm going to ask you about any illness or sickness that is not known or recognized in the community, including by medical or treatment providers.

28. Where do you usually get treatment for medical problems? Select all that apply.
   - ○ clinic/health center
   - ○ hospital
   - ○ mobile clinic
   - ○ community health worker
   - ○ traditional healer
   - ○ dispensary or pharmacy

29. Have you ever had an unusual illness with any of the following symptoms: Select all that apply. (READ ONLY SYMPTOMS)
   - ○ fever with headache and severe fatigue or weakness (encephalitis)
   - ○ fever with bleeding or bruising not related to injury (hemorrhagic fever)
   - ○ fever with cough and shortness of breath or difficulty breathing (SARI)
   - ○ fever with muscle aches, cough, or sore throat (ILI)
   - ○ fever with diarrhea or vomiting
   - ○ fever with rash
   - ○ persistent rash or sores on skin
   - ○ no (Skip to question 33)
   - ○ yes but, none of these symptoms-describe: ____________

30. Since this time last year, have you had any of these symptoms?
   - ○ yes
   - ○ no (Skip to question 33)

31. If yes, which ones? Select all that apply.
   - ○ fever with headache and severe fatigue or weakness (encephalitis)
   - ○ fever with bleeding or bruising not related to injury (hemorrhagic fever)
   - ○ fever with cough and shortness of breath or difficulty breathing (SARI)
   - ○ fever with muscle aches, cough, or sore throat (ILI)
   - ○ fever with diarrhea or vomiting
   - ○ fever with rash
   - ○ persistent rash or sores on skin
   - ○ yes but, none of these symptoms-describe: ____________

32. In your opinion, when you were sick, what caused this sickness? Select all that apply.
   - ○ contact with sick people
   - ○ contact with wild animals
   - ○ contact with other animals
   - ○ bad food or water
   - ○ bad spirits/witchcraft
   - ○ wound or injury
   - ○ I don't know
   - ○ other: ____________

33. Since this time last year, have any of the people you lived with had any of these symptoms?
   - ○ yes
   - ○ no (Skip to question 36)

34. If yes, which ones? Select all that apply.
   - ○ fever with headache and severe fatigue or weakness (encephalitis)
   - ○ fever with bleeding or bruising not related to injury (hemorrhagic fever)
   - ○ fever with cough and shortness of breath or difficulty breathing (SARI)
   - ○ fever with muscle aches, cough, or sore throat (ILI)
   - ○ fever with diarrhea or vomiting
   - ○ fever with rash
   - ○ persistent rash or sores on skin
   - ○ yes but, none of these symptoms-describe: ____________

35. Since this time last year, did anyone you lived with die from this illness?
   - ○ yes
   - ○ no

153896-108604

Participant ID ____________
(For reference only)

Movement Section

In this section, I'm going to ask you about any travel you have done since this time last year.

36. Have you traveled since this time last year?
If answer is no, skip to the next section.
- ○ yes
- ○ no

37. Where have you traveled since this time last year? Anywhere else?
Provide details, such as name of town, nearest (or most frequent) well known place if unknown by interviewer (to be linked to GPS coordinates later)
Collect up to 6 locations.
Interviewer: Probe for landmarks or nearest known site if area unknown. GPS coordinates to be identified and entered after completion of interview.

Village/Town/City ____________ District ____________ Province/State ____________
Latitude ____________ Longitude ____________
Notes: ____________

Village/Town/City ____________ District ____________ Province/State ____________
Latitude ____________ Longitude ____________
Notes: ____________

Village/Town/City ____________ District ____________ Province/State ____________
Latitude ____________ Longitude ____________
Notes: ____________

Village/Town/City ____________ District ____________ Province/State ____________
Latitude ____________ Longitude ____________
Notes: ____________

Village/Town/City ____________ District ____________ Province/State ____________
Latitude ____________ Longitude ____________
Notes: ____________

Village/Town/City ____________ District ____________ Province/State ____________
Latitude ____________ Longitude ____________
Notes: ____________

If there are more than six locations check here. ○
Do not collect additional location information.

38. Why have you traveled?
Select all that apply.
- ○ work
- ○ visit family
- ○ moved
- ○ religious reasons
- ○ holiday/vacation
- ○ go to hospital/seek medical care
- ○ go to market
- ○ other: ____________

153897-108604

Participant ID ____________
(For reference only)

Animal Contact Section

In this section, I'm going to ask you about the animals in your life.

If answered "no" under the "In your lifetime" column, then no answer is required under the "Since this time last year" column.

Question	In your lifetime...	Since this time last year...
39. Has an animal lived as a pet in or near your dwelling?	○ yes ○ no	○ yes ○ no
40. Have you handled live animals?	○ yes ○ no	○ yes ○ no
41. Have you raised live animals?	○ yes ○ no	○ yes ○ no
42. Have you shared a water source with animals for washing?	○ yes ○ no ○ don't know	○ yes ○ no ○ don't know
43. Have you seen animal feces in or near food before you have eaten it?	○ yes ○ no	○ yes ○ no
44. Have you eaten food after an animal has touched or damaged it? (Example: chew marks or	○ yes ○ no ○ don't know	○ yes ○ no ○ don't know
45. Do any animals come inside the dwelling where you live?	○ yes ○ no	○ yes ○ no
46. Have you cooked or handled meat, organs or blood from a recently killed animal?	○ yes ○ no	○ yes ○ no
47. Have you eaten raw or undercooked meat or organs or blood?	○ yes ○ no	○ yes ○ no
48. Have you eaten an animal that you knew was not well/sick?	○ yes ○ no ○ don't know	○ yes ○ no ○ don't know
49. Have you found a dead animal and collected it to eat or share?	○ yes ○ no	○ yes ○ no
50. Have you found a dead animal and collected it to sell it?	○ yes ○ no	○ yes ○ no
51. Have you been scratched or bitten by an animal?	○ yes ○ no	○ yes ○ no

52. The last time you were scratched, bitten or cut yourself while butchering or slaughtering, what did you do?
Select all that apply.

- ○ let someone else take over
- ○ wash wound with soap and water
- ○ rinse wound with water
- ○ bandage wound
- ○ visit doctor
- ○ nothing - kept working
- ○ never butcher or slaughter

153898-108604

Animal Contact Section

53. Are there any risks associated with slaughtering or butchering when you have an open wound?

Interviewer: Do not read responses.

- ◯ no
- ◯ yes, but I don't know what they are
- ◯ yes, it can make you sick
- ◯ yes, it can poison you
- ◯ yes, it can infect you with a disease
- ◯ don't know
- ◯ other: ______________________________

	In your lifetime...	Since this time last year...
54. Have you slaughtered an animal?	◯ yes<br>◯ no	◯ yes<br>◯ no
55. Have you hunted or trapped an animal?<br>(If answered "yes" to "Since this time last year" also ask hunter questionnaire)	◯ yes<br>◯ no	◯ yes<br>◯ no

56. Interviewer: Circle all headings where “yes” was answered in the "Since this time last year" questions above.
Then ask which animals/mammals for each “yes” category.
Select all that apply.

	pet (39)	handled (40)	raised (41)	feces in or near food (43)	in house (45)	cooked/ handled (46)	eaten raw/ under cooked (47)	eaten sick (48)	found dead (49/50)	scratched/bitten (51)	slaugh-tered (54)	hunted/ trapped (55)
rodents/shrews	◯	◯	◯	◯	◯	◯	◯	◯	◯	◯	◯	◯
bats	◯	◯	◯	◯	◯	◯	◯	◯	◯	◯	◯	◯
non-human primates	◯	◯	◯	◯	◯	◯	◯	◯	◯	◯	◯	◯
birds	◯	◯	◯	◯	◯	◯	◯	◯	◯	◯	◯	◯
carnivores	◯	◯	◯	◯	◯	◯	◯	◯	◯	◯	◯	◯
ungulates	◯	◯	◯	◯	◯	◯	◯	◯	◯	◯	◯	◯
pangolins	◯	◯	◯	◯	◯	◯	◯	◯	◯	◯	◯	◯
poultry/other fowl	◯	◯	◯	◯	◯	◯	◯	◯	◯	◯	◯	◯
goats/sheep	◯	◯	◯	◯	◯	◯	◯	◯	◯	◯	◯	◯
camels	◯	◯	◯	◯	◯	◯	◯	◯	◯	◯	◯	◯
swine	◯	◯	◯	◯	◯	◯	◯	◯	◯	◯	◯	◯
cattle/buffalo	◯	◯	◯	◯	◯	◯	◯	◯	◯	◯	◯	◯
dogs	◯	◯	◯	◯	◯	◯	◯	◯	◯	◯	◯	◯
cats	◯	◯	◯	◯	◯	◯	◯	◯	◯	◯	◯	◯

57. Are you worried about diseases or disease outbreaks in live animals in your local market? ◯ yes ◯ no

END OF MAIN QUESTIONNAIRE

153899-108604

EIDITH
V 1.1

0 1 2 3 4 5 6 7 8 9
0 1 2 3 4 5 6 7 8 9
0 1 2 3 4 5 6 7 8 9
0 1 2 3 4 5 6 7 8 9
0 1 2 3 4 5 6 7 8 9
0 1 2 3 4 5 6 7 8 9

Add Human Questionnaire Form ID

Participant ID ____________________
(For reference only)

1. Do you live on site?
   - yes
   - no

2. To the best of your knowledge, how many people work at this site? Select one option.
   - <10
   - 10-100
   - 101-1000
   - 1001-10,000
   - >10,000

3. How long have you worked here? Select one option.
   - <1 month
   - 1 month - 1 year
   - >1 year - 5 years
   - >5 years

4. Which animals are raised here? Select all that apply.
   - rodents/shrews
   - bats
   - non-human primates
   - birds
   - carnivores
   - ungulates
   - pangolins
   - poultry/other fowl
   - goats/sheep
   - camels
   - swine
   - cattle/buffalo
   - dogs
   - cats

5. How are live animals stored at night? Select all that apply.
   - multiple species in one enclosure
   - individual species in one enclosure
   - both multiple and individual species in enclosures

6. Is there a quarantine period for new animals?
   - yes
   - no

7. Is there on-site food production?
   - yes
   - no

8. If yes, who pays for the cost to grow the food crops?
   - the company
   - the workers

9. Is there meat available for consumption?
   - yes
   - no

150843-451831

10. If yes, where does the meat come from?
    Select all that apply.
    - farmed onsite
    - farmed and purchased from nearby local communities
    - purchased from wholesale market
    - locally caught/hunted
    - bought frozen
    - don't know

11. Is it possible to consume bushmeat/wild animal meat on or near the site?
    - yes
    - no

12. Do you have special protective equipment (Example: shoes, masks, gloves) only worn at work?
    - yes
    - no

13. If yes, which protective equipment?
    Select all that apply.
    - shoes/boots
    - mask
    - clothes
    - gloves
    - gown/apron

14. When do you use protective equipment?
    Select all that apply.
    - handling animals
    - slaughter
    - butcher
    - always on at work
    - other: ____________________

15. Do you always use disinfectant to clean?
    - yes
    - no

16. If yes, do you always use disinfectants to clean the following:
    Select all that apply.
    - animal enclosures
    - food bins
    - counter tops
    - slaughtering/butchering equipment
    - hands
    - special protective equipment
    - floors

17. How often are the animal enclosures cleaned?
    Select one option.
    - daily
    - weekly
    - monthly
    - as needed
    - never

18. When slaughtering/butchering animals, what happens to the viscara (blood, organs, skin, sinews, etc)?
    Select all that apply.
    - sell
    - throw into refuse bin
    - throw into the street/gutter
    - take home to eat
    - feed to animals
    - no onsite slaughter

19. Is there a designated area for the disposal of animal waste?
    - yes
    - no

20. If yes, do people use the dedicated area for animal waste?
    - yes
    - no

150844-451831

21. Since this time last year, have the animals received veterinary care?
   - yes
   - no

22. Since this time last year, has an animal health official inspected your animals?
   - yes
   - no

23. What do you do when an animal gets sick? Select all that apply.
   - kill the animal and dispose of the carcass
   - kill the animal and sell it
   - sell the live animal for discounted price
   - nothing different
   - get veterinary care
   - report to authorities
   - other: ____________________

24. Since this time last year, has anyone quarantined or destroyed your animals because of infection or disease?
   - yes
   - no

25. If yes, which animals? Select all that apply.
   - rodents/shrews
   - bats
   - non-human primates
   - birds
   - carnivores
   - ungulates
   - pangolins
   - poultry/other fowl
   - goats/sheep
   - camels
   - swine
   - cattle/buffalo
   - dogs
   - cats

26. Since this time last year, has there been a disease outbreak among any raised animals or livestock?
   - yes
   - no

27. If yes, which animals? (Indicate the percentage that died during the outbreak.) Select all that apply.

	1-25%	26-50%	51-75%	76-100%	don't know
rodents/shrews					
bats					
non-human primates					
birds					
carnivores					
ungulates					
pangolins					
poultry/other fowl					
goats/sheep					
camels					
swine					
cattle/buffalo					
dogs					
cats					

28. Do any animals raid or destroy food supplies?
- yes
- no

29. If yes, what animals? Select all that apply.
- rodents/shrews
- bats
- non-human primates
- birds
- carnivores
- ungulates
- pangolins
- poultry/other fowl
- goats/sheep
- camels
- swine
- cattle/buffalo
- dogs
- cats

30. What is done to stop animals from raiding or destroying food supplies? Select all that apply.
- barriers around fields
- barriers on individual trees
- fire
- poison
- traps
- shooting
- loud sounds
- domestic/guardian animals
- flooding
- chasing animals out
- nothing

Notes:

150846-451831

Crop Production Module

EIDITH
V 1.0

0 1 2 3 4 5 6 7 8 9
0 1 2 3 4 5 6 7 8 9
0 1 2 3 4 5 6 7 8 9
0 1 2 3 4 5 6 7 8 9
0 1 2 3 4 5 6 7 8 9
0 1 2 3 4 5 6 7 8 9

Add Human Questionnaire Form ID

Participant ID ______________________
(For reference only)

1. Do you live on the work site?
   - ○ yes
   - ○ no

2. To the best of your knowledge, how many people work at this site?
   Select one option.
   - ○ <10
   - ○ 10-100
   - ○ 101-1000
   - ○ 1001-10,000
   - ○ >10,000

3. How long have you worked at this site?
   Select one option.
   - ○ <1 month
   - ○ 1 month - 1 year
   - ○ >1 year - 5 years
   - ○ >5 years

4. Which crops are at this site?
   Select all that apply.
   - ○ coffee/tea/cocoa plants
   - ○ fruit/nut trees
   - ○ oil tree plantation
   - ○ oil seed crops
   - ○ dry grains
   - ○ sugar
   - ○ vegetable/fruit crops
   - ○ pulses/legume
   - ○ fiber
   - ○ forages
   - ○ cover crops
   - ○ fallow fields
   - ○ rubber

5. Does the farm use manure, guano or night soil to fertilizer the crops?
   - ○ yes
   - ○ no

6. If yes, which types?
   Select all that apply.
   - ○ poultry/other fowl
   - ○ camel
   - ○ swine
   - ○ cattle/buffalo
   - ○ bird guano
   - ○ bat guano
   - ○ night soil

7. Is there meat available for the consumption?
   - ○ yes
   - ○ no

8. If yes, where does the meat come from?
   Select all that apply.
   - ○ farmed onsite
   - ○ farmed and purchased from nearby local communities
   - ○ purchased from wholesale market
   - ○ locally caught/hunted
   - ○ bought frozen
   - ○ don't know

9. Is it possible to consume bushmeat/wild animal meat on or near the site?
   - ○ yes
   - ○ no

103833-711174

10. Is there a designated area for rubbish, including animal waste from slaughter/butcher and animal excrement?
- ○ yes
- ○ no

11. If yes, do people use the designated location for rubbish?
- ○ yes
- ○ no

12. Do any animals raid food supplies or destroy crops?
- ○ yes
- ○ no

13. If yes, which animals? Select all that apply.
- ○ rodents/shrews
- ○ bats
- ○ non-human primates
- ○ birds
- ○ carnivores
- ○ ungulates
- ○ pangolins
- ○ poultry/other fowl
- ○ goats/sheep
- ○ camels
- ○ swine
- ○ cattle/buffalo
- ○ dogs
- ○ cats

14. What is done to stop animals from raiding or destroying food supplies? Select all that apply.
- ○ barriers around fields
- ○ barriers on individual trees
- ○ fire
- ○ poison
- ○ traps
- ○ shooting
- ○ loud sounds
- ○ domestic/guardian animals
- ○ flooding
- ○ chasing animals out
- ○ nothing

Extractive Industry Module

EIDITH
V 1.1

0 1 2 3 4 5 6 7 8 9
0 1 2 3 4 5 6 7 8 9
0 1 2 3 4 5 6 7 8 9
0 1 2 3 4 5 6 7 8 9
0 1 2 3 4 5 6 7 8 9
0 1 2 3 4 5 6 7 8 9

Add Human Questionnaire Form ID

Participant ID ____________________
(For reference only)

1. What type of work or industry is conducted here?
   Select one option.
   - ◯ underground mining (by shafts or tunnels)
   - ◯ open surface mining
   - ◯ hydraulic mining (high pressure water)
   - ◯ gathering, panning, or collecting
   - ◯ oil well/gas field
   - ◯ logging
   - ◯ other: ____________________

2. What product(s) are extracted?
   Select one option.
   - ◯ coal
   - ◯ coltan
   - ◯ diamond or other gemstone
   - ◯ tin
   - ◯ gold/silver
   - ◯ lead
   - ◯ oil/gas
   - ◯ timber/plant
   - ◯ electricity
   - ◯ other: ____________

3. Do you live on the work site?
   - ◯ yes
   - ◯ no

4. To the best of your knowledge, how many people work at this site?
   Select one option.
   - ◯ <10
   - ◯ 10-100
   - ◯ 101-1000
   - ◯ 1001-10,000
   - ◯ >10,000

5. How long have you worked at this site?
   Select one option.
   - ◯ <1 month
   - ◯ 1 month - 1 year
   - ◯ >1 year - 5 years
   - ◯ >5 years

6. Is there on-site food production?
   - ◯ yes
   - ◯ no

7. If yes, who pays for the cost to grow the food crops?
   - ◯ the company
   - ◯ the workers

8. Is there meat available for consumption?
   - ◯ yes
   - ◯ no

104091-367194

9. If yes, where does the meat come from?
   Select all that apply.
   - farmed onsite
   - farmed and purchased from nearby local communities
   - purchased from wholesale market
   - locally caught/hunted
   - bought frozen
   - don't know

10. Is it possible to consume bushmeat/wild animal meat on or near the site?
    - yes
    - no

11. Is there a designated area for rubbish, including animal waste from slaughter/butcher and animal excrement?
    - yes
    - no

12. If yes, do people use the designated location for rubbish?
    - yes
    - no

13. Do any animals raid food supplies or destroy crops?
    - yes
    - no

14. If yes, which animals?
    Select all that apply.
    - rodents/shrews
    - bats
    - non-human primates
    - birds
    - carnivores
    - ungulates
    - pangolins
    - poultry/other fowl
    - goats/sheep
    - camels
    - swine
    - cattle/buffalo
    - dogs
    - cats

15. What is done to stop animals from raiding or destroying food supplies?
    Select all that apply.
    - barriers around fields
    - barriers on individual trees
    - fire
    - poison
    - traps
    - shooting
    - loud sounds
    - domestic/guardian animals
    - flooding
    - chasing animals out
    - nothing

Hunter Module

EIDITH

V 1.1

0	1	2	3	4	5	6	7	8	9
0	1	2	3	4	5	6	7	8	9
0	1	2	3	4	5	6	7	8	9
0	1	2	3	4	5	6	7	8	9
0	1	2	3	4	5	6	7	8	9
0	1	2	3	4	5	6	7	8	9

Add Human Questionnaire Form ID

Participant ID ____________________

(For reference only)

1. What animals have you hunted since this time last year?
Select all that apply.

- ○ rodents/shrews
- ○ bats
- ○ non-human primates
- ○ birds
- ○ carnivores
- ○ ungulates
- ○ pangolins

2. Since this time last year, what methods have you used to hunt/trap animals?
Select all that apply.

- ○ snare
- ○ bow
- ○ hands
- ○ gun
- ○ machete
- ○ knife
- ○ net
- ○ cage
- ○ trap
- ○ other: ______________

3. What is the purpose of your trapping or hunting?
Select all that apply.

	for consumption at home	for use of animal products at home	for sale for consump -tion	for sale alive at market	for sale of animal products	live trapping of nuisance animals for translocation	culling of nuisance animals
rodents/shrews	○	○	○	○	○	○	○
bats	○	○	○	○	○	○	○
non-human primates	○	○	○	○	○	○	○
birds	○	○	○	○	○	○	○
carnivores	○	○	○	○	○	○	○
ungulates	○	○	○	○	○	○	○
pangolins	○	○	○	○	○	○	○

Since this time last year, when you hunt or trap:

4. Are you exposed to blood?
- ○ yes
- ○ no

5. Have you been scratched or bitten?
- ○ yes
- ○ no

6. Since this time last year, have you seen an outbreak of dead wild animals?
- ○ yes
- ○ no

7. If yes, which wild animals?
Select all that apply.

- ○ rodents/shrews
- ○ bats
- ○ non-human primates
- ○ birds
- ○ carnivores
- ○ ungulates
- ○ pangolins

104897-690914

8. What do you do when you find an animal dead (not in a trap or shot by another hunter)?
Select all that apply.

- ◯ touch it to see if it is still fresh
- ◯ butcher in the forest
- ◯ smoke or cook in the forest
- ◯ take home to prepare
- ◯ bury it
- ◯ report it to authorities
- ◯ take it to sell it
- ◯ nothing
- ◯ other: ________________

9. How do you transport a dead animal, if you take it?
Select all that apply.

- ◯ not wrapped
- ◯ wrapped in leaves or other natural material
- ◯ wrapped in plastic
- ◯ in a bag
- ◯ in a basket

10. Do you have special protective equipment (Example: shoes, masks, gloves)?

- ◯ yes
- ◯ no

11. If yes, which protective equipment?
Select all that apply.

- ◯ shoes/boots
- ◯ mask
- ◯ clothes
- ◯ gloves
- ◯ gown/apron

12. When do you use protective equipment?
Select all that apply.

- ◯ handling animals
- ◯ slaughter
- ◯ butcher
- ◯ always on at work
- ◯ other: ________________

Market & Value Chain Module

EIDITH

V 1.0

0 1 2 3 4 5 6 7 8 9
0 1 2 3 4 5 6 7 8 9
0 1 2 3 4 5 6 7 8 9
0 1 2 3 4 5 6 7 8 9
0 1 2 3 4 5 6 7 8 9
0 1 2 3 4 5 6 7 8 9

Add Human Questionnaire Form ID

Participant ID ____________________

(For reference only)

1. Do you live on the work site?
   - yes
   - no

2. To the best of your knowledge, how many people work at this site?
   Select one option.
   - <10
   - 10-50
   - 51-100
   - 101-1000
   - >1001

3. How long have you worked at this market?
   Select one option.
   - <1 month
   - 1 month - 1 year
   - >1 year - 5 years
   - >5 years

4. What animals are you selling today?
   Select all that apply.
   - rodents/shrews
   - bats
   - non-human primates
   - birds
   - carnivores
   - ungulates
   - pangolins
   - poultry/other fowl
   - goats/sheep
   - camels
   - swine
   - cattle/buffalo
   - dogs
   - cats

5. Have you sold a live animal today?
   - yes
   - no

6. Who buys the live animals you sell?
   Select all that apply.
   - customer for home use
   - restaurants/hotels
   - customer who resells at another market
   - other: ____________________

7. Where do the animals come from?
   Select all that apply.
   - farmed and/or purchased from nearby local communities
   - wholesale live animal market
   - locally caught/hunted
   - other: ____________________

8. How do the live animals get to the market?
   Select all that apply.
   - transport truck
   - car
   - motorbike
   - cart
   - delivered by hunter
   - public bus
   - other: ____________________

151798-452339

9. How are live animals stored at night?
   Select all that apply.
   - multiple species in one enclosure
   - individual species in one enclosure
   - both multiple and individual species in enclosures

10. How long do you keep alive animals before you sell them? (in hours) ___________

11. What do you do when an animal gets sick?
    Select all that apply.
    - kill the animal and dispose of the carcass
    - kill the animal and sell it
    - sell the live animal for discounted price
    - nothing different
    - get veterinary care
    - report to authorities
    - other: ______________________________

12. Do you have special protective equipment (Example: shoes, masks, gloves) only worn at work?
    - yes
    - no

13. If yes, which protective equipment?
    Select all that apply.
    - shoes/boots
    - mask
    - clothes
    - gloves
    - gown/apron

14. When do you use protective equipment?
    Select all that apply.
    - handling animals
    - slaughter
    - butcher
    - always on at work
    - other: ______________________

15. Is protective equipment used every time an animal is handled?
    - yes
    - no

16. When butchering animals, what happens to the refuse (blood, organs, skin, sinews, etc)?
    Select all that apply.
    - sell
    - throw into refuse bin
    - throw into the street/gutter
    - take home to eat
    - feed to animals
    - no onsite slaughter

17. Do you always use disinfectant to clean?
    - yes
    - no

18. If yes, do you always use disinfectants to clean the following:
    Select all that apply.
    - animal enclosures
    - food bins
    - counter tops
    - slaughtering/butchering equipment
    - hands
    - special protective equipment
    - floors

151799-452339

19. How often are the animal enclosures cleaned?
    Select one option.
    - daily
    - weekly
    - monthly
    - as needed
    - never

20. Is there a designated area for rubbish, including animal waste from slaughter/ butcher and animal excrement?
    - yes
    - no

21. If yes, do people use the dedicated area for rubbish?
    - yes
    - no

22. How often does the market close?
    Select one option.
    - once per week
    - once every 2 weeks
    - once per month
    - as needed
    - only operates 1-5 days per week
    - never

23. Since this time last year, has an animal health official inspected your animals?
    - yes
    - no

24. Since this time last year, has anyone destroyed your animals because of infection or disease?
    - yes
    - no

25. If yes, which animals?
    Select all that apply.
    - rodents/shrews
    - bats
    - non-human primates
    - birds
    - carnivores
    - ungulates
    - pangolins
    - poultry/other fowl
    - goats/sheep
    - camels
    - swine
    - cattle/buffalo
    - dogs
    - cats

26. What is done to stop animals from raiding or destroying food supplies?
    Select all that apply.
    - barriers around fields
    - barriers on individual trees
    - fire
    - poison
    - traps
    - shooting
    - loud sounds
    - domestic/guardian animals
    - flooding
    - chasing animals out
    - nothing

151800-452339

Temporary Settlement Module

EIDITH V 1.0

0 1 2 3 4 5 6 7 8 9
0 1 2 3 4 5 6 7 8 9
0 1 2 3 4 5 6 7 8 9
0 1 2 3 4 5 6 7 8 9
0 1 2 3 4 5 6 7 8 9
0 1 2 3 4 5 6 7 8 9

Add Human Questionnaire Form ID

Participant ID ____________________
(For reference only)

1. What is your nationality? ____________________

2. How long have you lived at this settlement?
   Select one option.
   - ○ <1 week
   - ○ 1-4 weeks
   - ○ 1-5 months
   - ○ 6-11 months
   - ○ > 1 year
   - ○ entire life

3. To the best of your knowledge, how many people live at this site?
   Select one option.
   - ○ <10
   - ○ 10-100
   - ○ 101-1000
   - ○ 1001-10,000
   - ○ >10,000

4. Are all the people living here from this country?
   - ○ yes
   - ○ no

5. Why did you settle here?
   Select all that apply.
   - ○ job/work (voluntary relocation)
   - ○ family (voluntary relocation)
   - ○ marriage (voluntary relocation)
   - ○ conflict (forced relocation)
   - ○ dispossession of previous home (forced relocation)
   - ○ natural disaster (forced relocation)

6. Is there on-site food production?
   - ○ yes
   - ○ no

7. Is there meat available for consumption?
   - ○ yes
   - ○ no

8. If yes, where does the meat come from?
   Select all that apply.
   - ○ farmed onsite
   - ○ farmed and purchased from nearby local communities
   - ○ purchased from wholesale market
   - ○ locally caught/hunted
   - ○ bought frozen
   - ○ don't know

9. Is it possible to consume bushmeat/wild animal meat on or near the site?
   - ○ yes
   - ○ no

152291-810424

10. Which animals are available for consumption?
Select all that apply.
- ◯ rodents/shrews
- ◯ bats
- ◯ non-human primates
- ◯ birds
- ◯ carnivores
- ◯ ungulates
- ◯ pangolins
- ◯ poultry/other fowl
- ◯ goats/sheep
- ◯ camels
- ◯ swine
- ◯ cattle/buffalo
- ◯ dogs
- ◯ cats

11. Is there a designated area for rubbish, including animal waste from slaughter/butcher and animal excrement?
- ◯ yes
- ◯ no

12. If yes, do people use the designated location for rubbish?
- ◯ yes
- ◯ no

13. Do any animals raid food supplies or destroy crops?
- ◯ yes
- ◯ no

14. If yes, which animals?
Select all that apply.
- ◯ rodents/shrews
- ◯ bats
- ◯ non-human primates
- ◯ birds
- ◯ carnivores
- ◯ ungulates
- ◯ pangolins
- ◯ poultry/other fowl
- ◯ goats/sheep
- ◯ camels
- ◯ swine
- ◯ cattle/buffalo
- ◯ dogs
- ◯ cats

15. What is done to stop animals from raiding or destroying food supplies?
Select all that apply.
- ◯ barriers around fields
- ◯ barriers on individual trees
- ◯ fire
- ◯ poison
- ◯ traps
- ◯ shooting
- ◯ loud sounds
- ◯ domestic/guardian animals
- ◯ flooding
- ◯ chasing animals out
- ◯ nothing

Tourism Module

EIDITH
V 1.0

0 1 2 3 4 5 6 7 8 9
0 1 2 3 4 5 6 7 8 9
0 1 2 3 4 5 6 7 8 9
0 1 2 3 4 5 6 7 8 9
0 1 2 3 4 5 6 7 8 9
0 1 2 3 4 5 6 7 8 9

Add Human Questionnaire Form ID

Participant ID ______________________
(For reference only)

1. What is your nationality? ______________________

2. List all the countries you have visited in the past month.

______________________ ______________________
______________________ ______________________
______________________ ______________________

3. How long have you been at the current location?
Select one option.

- ◯ 1 day
- ◯ 2-7 days
- ◯ 8-14 days
- ◯ > 2 weeks

4. Which activities did you undertake on this trip?
Select all that apply.

- ◯ wildlife tourism
- ◯ cave tourism
- ◯ camping
- ◯ hunting
- ◯ trekking in natural areas
- ◯ religious pilgrimage
- ◯ visiting wildlife market
- ◯ other: ______________________

5. What animals have you eaten during this trip?
Select all that apply.

- ◯ rodents/shrews
- ◯ bats
- ◯ non-human primates
- ◯ birds
- ◯ carnivores
- ◯ ungulates
- ◯ pangolins
- ◯ poultry/other fowl
- ◯ goats/sheep
- ◯ camels
- ◯ swine
- ◯ cattle/buffalo
- ◯ dogs
- ◯ cats
- ◯ unknown
- ◯ none

Wildlife Restaurant Module

V 1.0

0 1 2 3 4 5 6 7 8 9
0 1 2 3 4 5 6 7 8 9
0 1 2 3 4 5 6 7 8 9
0 1 2 3 4 5 6 7 8 9
0 1 2 3 4 5 6 7 8 9
0 1 2 3 4 5 6 7 8 9

Add Human Questionnaire Form ID

Participant ID ____________________
(For reference only)

1. Do you live on site?
   - yes
   - no

2. To the best of your knowledge, how many people work at this site?
   - <10
   - 11-50
   - 51-100
   - 101-1000
   - >1001

3. How long have you worked here?
   Select one option.
   - <1 month
   - 1 month - 1 year
   - >1 year - 5 years
   - >5 years

4. What wild animals are on the menu today?
   Select all that apply.
   - rodents/shrews
   - bats
   - non-human primates
   - birds
   - carnivores
   - ungulates
   - pangolins

5. Since this time last year, have you had live animals on site?
   - yes
   - no

6. If yes, where do the animals come from?
   Select all that apply.
   - farmed and/or purchased from nearby local communities
   - wholesale live animal market
   - locally caught/hunted
   - other: ____________________

7. How do the live animals get to the restaurant?
   Select all that apply.
   - transport truck
   - car
   - motorbike
   - cart
   - delivered by hunter
   - public bus
   - brought in by customer
   - other: ____________________

107004-721313

8. How are live animals stored at night?
   Select all that apply.
   - multiple species in one enclosure
   - individual species in one enclosure
   - both multiple and individual species in enclosures

9. Are live animals slaughtered at the restaurant?
   - yes
   - no

10. Do you have special protective equipment (Example: shoes, masks, gloves) only worn at work?
    - yes
    - no

11. If yes, which protective equipment?
    Select all that apply.
    - shoes/boots
    - mask
    - clothes
    - gloves
    - gown/apron

12. When do you use protective equipment?
    Select all that apply.
    - handling animals
    - slaughter
    - butcher
    - always on at work
    - other: ______________________

13. Do you always use disinfectant to clean?
    - yes
    - no

14. If yes, do you always use disinfectants to clean the following:
    Select all that apply.
    - animal enclosures
    - food bins
    - counter tops
    - slaughtering/butchering equipment
    - hands
    - special protective equipment
    - floors

15. Is there a designated area for rubbish, including animal waste from slaughter/butcher and animal excrement?
    - yes
    - no

16. If yes, do people use the dedicated location for rubbish?
    - yes
    - no

107005-721313

17. Do any animals raid food or destroy supplies?
- yes
- no

18. If yes, which animals? Select all that apply.
- rodents/shrews
- bats
- non-human primates
- birds
- carnivores
- ungulates
- pangolins
- poultry/other fowl
- goats/sheep
- camels
- swine
- cattle/buffalo
- dogs
- cats

19. What is done to stop animals from raiding or destroying food supplies? Select all that apply.
- barriers around fields
- barriers on individual trees
- fire
- poison
- traps
- shooting
- loud sounds
- domestic/guardian animals
- flooding
- chasing animals out
- nothing

EIDITH
V 1.0

0 1 2 3 4 5 6 7 8 9
0 1 2 3 4 5 6 7 8 9
0 1 2 3 4 5 6 7 8 9
0 1 2 3 4 5 6 7 8 9
0 1 2 3 4 5 6 7 8 9
0 1 2 3 4 5 6 7 8 9

Add Human Questionnaire Form ID

Participant ID ____________________
(For reference only)

1. Do you live on site?
   - yes
   - no

2. To the best of your knowledge, how many people work at this site? Select one option.
   - <10
   - 11-50
   - 51-100
   - 101-1000
   - >1001

3. How long have you worked here? Select one option.
   - <1 month
   - 1 month - 1 year
   - >1 year - 5 years
   - >5 years

4. What species do you work with? Select all that apply.
   - rodents/shrews
   - bats
   - non-human primates
   - birds
   - carnivores
   - ungulates
   - pangolins
   - poultry/other fowl
   - goats/sheep
   - camels
   - swine
   - cattle/buffalo
   - dogs
   - cats

5. Do you have special protective equipment (Example: shoes, masks, gloves) only worn at work?
   - yes
   - no

6. If yes, which protective equipment? Select all that apply.
   - shoes/boots
   - mask
   - clothes
   - gloves
   - gown/apron

7. When do you use protective equipment? Select all that apply.
   - handling animals
   - slaughter
   - butcher
   - always on at work
   - other: ____________________

107585-551187

8. Do you always use disinfectant to clean?
   - yes
   - no

9. If yes, do you always use disinfectants to clean the following:
   Select all that apply.
   - animal enclosures
   - food bins
   - counter tops
   - slaughtering/butchering equipment
   - hands
   - special protective equipment
   - floors

10. How often are the animal enclosures cleaned?
    Select one option.
    - daily
    - weekly
    - monthly
    - as needed
    - never

11. Is there a designated area for the disposal of animal waste?
    - yes
    - no

12. If yes, do people use the dedicated area for animal waste?
    - yes
    - no

13. Is there a quarantine period for new animals?
    - yes
    - no

14. Is there a preventative medicine program for the animals?
    - yes
    - no

15. Do any animals raid food or destroy supplies?
    - yes
    - no

16. If yes, which animals?
    Select all that apply.
    - rodents/shrews
    - bats
    - non-human primates
    - birds
    - carnivores
    - ungulates
    - pangolins
    - poultry/other fowl
    - goats/sheep
    - camels
    - swine
    - cattle/buffalo
    - dogs
    - cats

17. What is done to stop animals from raiding or destroying food supplies?
    Select all that apply.
    - barriers around fields
    - barriers on individual trees
    - fire
    - poison
    - traps
    - shooting
    - loud sounds
    - domestic/guardian animals
    - flooding
    - chasing animals out
    - nothing

107586-551187

Human Hospital & Clinic Module for Health Professionals

0	1	2	3	4	5	6	7	8	9
0	1	2	3	4	5	6	7	8	9
0	1	2	3	4	5	6	7	8	9
0	1	2	3	4	5	6	7	8	9
0	1	2	3	4	5	6	7	8	9
0	1	2	3	4	5	6	7	8	9

EIDITH
V 1.0

Add Human Questionnaire Form ID

Participant ID ______________________
(For reference only)

1. What are your human health activities?
   - hospital or clinic administrator
   - hospital or clinic custodial worker
   - hospital or clinic clinician or nurse (medicine specialty)
   - hospital or clinic clinician or nurse (surgery specialty)
   - mobile clinic
   - traditional healer
   - dispensary or pharmacy

2. Do you have special protective equipment (Example: shoes, masks, gloves) only worn at work?
   - yes
   - no

3. If yes, which protective equipment? Select all that apply.
   - shoes/boots
   - mask
   - clothes
   - gloves
   - gown/apron

4. Is protective equipment used every time you examine or collect specimens from a patient?
   - yes
   - no

5. Is the protective equipment cleaned, sterilized or discarded after each use?
   - yes
   - no

6. Do you always use disinfectants to clean equipment and hospital areas?
   - yes
   - no
