# Supplementary material for: Knowledge, attitudes, and practices associated with zoonotic disease transmission risk in North Sulawesi, Indonesia
Source: One Health Outlook. 2022 Jun 3;4:11. doi: 10.1186/s42522-022-00067-w (PMC9162794; doi:10.1186/s42522-022-00067-w)
Supplement: Supplementary file 2 — Additional file 2: S2 File. Table S1. Type of contact with wildlife and livestock of participants for quantitative data collection (n = 477). Table S2. Perception of the cause of sickness. Table S3. Logistic regression analysis on potential social economic factors contributed to wound treatment behavior. Table S4. Treatment seeking behavior of the study participants. Table S5. Self-reported unusual symptoms in the past year period. Table S6. Correlation between wildlife contacts and self-reported symptoms. [file 42522_2022_67_MOESM2_ESM.docx]

**S2 File. Supplementary Table**

Table S1. Type of contact with wildlife and livestock of participants for quantitative data collection (n=477). Respondents could choose more than one type of contacts for each taxa.

| Animal taxa | Village A (n = 154) | Village B (n = 112) | Village C  (n = 111) | Village D (n = 100) | Total (N = 477) | Activity | p-value Chi-square test |
| --- | --- | --- | --- | --- | --- | --- | --- |
| Rodents | **44 (29%)** | **83 (74%)** | **90 (81%)** | **77 (77%)** | **294 (62%)** | **Cooked/handled** | **< 0.001** |
|  | 1 (1%) | 0 (0%) | 0 (0%) | 0 (0%) | 1 (0%) | Eaten raw/undercooked | 1.000 |
|  | 0 (0%) | 0 (0%) | 0 (0%) | 1 (1%) | 1 (0%) | Eaten sick | 0.218 |
|  | **0 (0%)** | **0 (0%)** | **0 (0%)** | **2 (2%)** | **2 (0%)** | **Feces in or near food** | **0.040** |
|  | 1 (1%) | 0 (0%) | 0 (0%) | 0 (0%) | 1 (0%) | Found dead/collected | 1.000 |
|  | 0 (0%) | 1 (1%) | 0 (0%) | 0 (0%) | 1 (0%) | Handled | 0.679 |
|  | 19 (12%) | 10 (9%) | 12 (11%) | 7 (7%) | 48 (10%) | Hunted/trapped | 0.545 |
|  | **7 (5%)** | **55 (49%)** | **63 (57%)** | **9 (9%)** | **134 (28%)** | **In-house** | **<0.001** |
|  | 5 (3%) | 3 (3%) | 1 (1%) | 0 (0%) | 9 (2%) | Scratched/bitten | 0.232 |
|  | 13 (8%) | 4 (4%) | 3 (3%) | 3 (3%) | 23 (5%) | Slaughtered | 0.081 |
| Bats | **53 (34%)** | **86 (77%)** | **91 (82%)** | **63 (63%)** | **293 (61%)** | **Cooked/handled** | **<0.001** |
|  | 1 (1%) | 0 (0%) | 0 (0%) | 0 (0%) | 1 (0%) | Eaten raw/undercooked | 1.000 |
|  | 0 (0%) | 0 (0%) | 0 (0%) | 1 (1%) | 1 (0%) | Eaten sick | 0.220 |
|  | 0 (0%) | 1 (1%) | 0 (0%) | 1 (1%) | 2 (0%) | Handled | 0.449 |
|  | **22 (14%)** | **5 (5%)** | **8 (7%)** | **0 (0%)** | **35 (7%)** | **Hunted/trapped** | **<0.001** |
|  | 0 (0%) | 2 (2%) | 1 (1%) | 1 (1%) | 4 (1%) | In house | 0.529 |
|  | 3 (2%) | 1 (1%) | 0 (0%) | 0 (0%) | 4 (1%) | Scratched/bitten | 0.257 |
|  | **16 (10%)** | **2 (2%)** | **1 (1%)** | **1 (1%)** | **20 (4%)** | **Slaughtered** | **<0.001** |
| Swine | **98 (64%)** | **88 (79%)** | **95 (86%)** | **88 (88%)** | **369 (77%)** | **Cooked/handled** | **<0.001** |
|  | **12 (8%)** | **2 (2%)** | **2 (2%)** | **3 (3%)** | **19 (4%)** | **Eaten raw/undercooked** | **0.030** |
|  | 1 (1%) | 1 (1%) | 0 (0%) | 0 (0%) | 2 (0%) | Eaten sick | 1.000 |
|  | 0 (0%) | 2 (2%) | 0 (0%) | 0 (0%) | 2 (0%) | Feces in or near food | 0.149 |
|  | 2 (1%) | 0 (0%) | 0 (0%) | 0 (0%) | 2 (0%) | Found dead/collected | 0.254 |
|  | **0 (0%)** | **14 (13%)** | **1 (1%)** | **2 (2%)** | **17 (4%)** | **Handled** | **<0.001** |
|  | **4 (3%)** | **0 (0%)** | **0 (0%)** | **0 (0%)** | **4 (1%)** | **Hunted/trapped** | **0.018** |
|  | 5 (3%) | 3 (3%) | 4 (4%) | 4 (4%) | 16 (3%) | In house | 0.972 |
|  | **0 (0%)** | **14 (13%)** | **1 (1%)** | **2 (2%)** | **17 (4%)** | **Pets** | **<0.001** |
|  | **0 (0%)** | **14 (13%)** | **1 (1%)** | **3 (3%)** | **18 (4%)** | **Raised** | **<0.001** |
|  | 0 (0%) | 0 (0%) | 0 (0%) | 1 (1%) | 1 (0%) | Scratched/bitten | 0.210 |
|  | 4 (3%) | 1 (1%) | 2 (2%) | 3 (3%) | 10 (2%) | Slaughtered | 0.696 |
| Poultry | 139 (90%) | 100 (89%) | 100 (90%) | 91 (91%) | 430 (90%) | Cooked/handled | 0.985 |
|  | **2 (1%)** | **0 (0%)** | **0 (0%)** | **6 (6%)** | **8 (2%)** | **Eaten raw/undercooked** | **0.001** |
|  | **8 (5%)** | **3 (3%)** | **11 (10%)** | **11 (11%)** | **33 (7%)** | **Eaten sick** | **0.044** |
|  | **84 (55%)** | **104 (93%)** | **104 (94%)** | **64 (64%)** | **356 (75%)** | **Feces in or near food** | **<0.001** |
|  | 0 (0%) | 0 (0%) | 0 (0%) | 1 (1%) | 1 (0%) | Found dead/collected | 0.206 |
|  | 101 (66%) | 90 (80%) | 64 (58%) | 63 (63%) | 318 (67%) | Handled | **0.002** |
|  | 1 (1%) | 0 (0%) | 2 (2%) | 1 (1%) | 4 (1%) | Hunted/trapped | 0.576 |
|  | **135 (88%)** | **104 (93%)** | **110 (99%)** | **95 (95%)** | **444 (93%)** | **In house** | **0.004** |
|  | **100 (65%)** | **90 (80%)** | **65 (59%)** | **63 (63%)** | **318 (67%)** | **Pets** | **0.005** |
|  | **101 (66%)** | **90 (80%)** | **64 (58%)** | **62 (62%)** | **317 (67%)** | **Raised** | **0.002** |
|  | 5 (3%) | 5 (5%) | 1 (1%) | 4 (4%) | 15 (3%) | Scratched/bitten | 0.447 |
|  | **81 (53%)** | **85 (76%)** | **78 (70%)** | **45 (45%)** | **289 (61%)** | **Slaughtered** | **<0.001** |
| Dogs | **46 (30%)** | **71 (63%)** | **81 (73%)** | **47 (47%)** | **245 (51%)** | **Cooked/handled** | **<0.001** |
|  | 1 (1%) | 1 (1%) | 0 (0%) | 1 (1%) | 3 (1%) | Eaten raw/undercooked | 0.893 |
|  | 0 (0%) | 0 (0%) | 2 (2%) | 0 (0%) | 2 (0%) | Eaten sick | 0.096 |
|  | **1 (1%)** | **5 (5%)** | **7 (6%)** | **1 (1%)** | **14 (3%)** | **Feces in or near food** | **0.020** |
|  | **35 (23%)** | **60 (54%)** | **51 (46%)** | **40 (40%)** | **186 (39%)** | **Handled** | **<0.001** |
|  | 0 (0%) | 0 (0%) | 1 (1%) | 0 (0%) | 1 (0%) | Hunted/trapped | 0.438 |
|  | **49 (32%)** | **89 (80%)** | **96 (87%)** | **46 (46%)** | **280 (59%)** | **In house** | **<0.001** |
|  | **35 (23%)** | **61 (55%)** | **51 (46%)** | **38 (38%)** | **185 (39%)** | **Pets** | **<0.001** |
|  | **35 (23%)** | **60 (54%)** | **50 (45%)** | **41 (41%)** | **186 (39%)** | **Raised** | **<0.001** |
|  | 4 (3%) | 7 (6%) | 6 (5%) | 4 (4%) | 21 (4%) | Scratched/bitten | 0.519 |
|  | 4 (3%) | 3 (3%) | 5 (5%) | 4 (4%) | 16 (3%) | Slaughtered | 0.837 |
| Non human primate | 35 (23%) | 21 (19%) | 22 (20%) | 13 (13%) | 91 (19%) | Cooked/handled | 0.296 |
|  | 1 (1%) | 0 (0%) | 0 (0%) | 0 (0%) | 1 (0%) | Eaten raw/undercooked | 1.000 |
|  | **14 (9%)** | **0 (0%)** | **1 (1%)** | **1 (1%)** | **16 (3%)** | **Hunted/trapped** | **<0.001** |
|  | 1 (1%) | 1 (1%) | 0 (0%) | 0 (0%) | 2 (0%) | In house | 1.000 |
|  | **7 (5%)** | **0 (0%)** | **0 (0%)** | **1 (1%)** | **8 (2%)** | **Slaughtered** | **0.006** |

**Table S2. Perception of the cause of sickness**

| Perception of cause of sickness* | (N=477) |
| --- | --- |
| Contact with sick people | 50 (10%) |
| Contact with wild animals | 6 (1%) |
| Bad food or water | 137 (29%) |
| Bad spirits/witchcraft | 3 (1%) |
| Wound or injury | 22 (5%) |
| Do not know | 114 (24%) |
| Other | 291 (61%) |

*Participants could choose more than one answer.

**Table S3. Logistic regression analysis on potential social economic factors contributed to wound treatment behavior.**

|  | Treated scratched/bitten (n = 413)* Adj-R2 = 0.07 | | |
| --- | --- | --- | --- |
|  | OR | 95% CI | p value |
| **Gender** |  |  |  |
| Female | Ref |  |  |
| Male | 1.28 | 0.77 – 2.14 | 0.34 |
| **Age** |  |  |  |
| Under 24 | Ref |  |  |
| 25 to 54 | 1.82 | 0.78 - | 0.17 |
| over 55 | 1.48 | 0.59 - | 0.40 |
| **Highest education (participant)** |  |  |  |
| None + primary school | Ref |  |  |
| Secondary school + College/university | 1.24 | 0.75 – 2.04 | 0.39 |
| **Highest education (participant’s mother)** |  |  |  |
| None + primary school | Ref |  |  |
| Secondary school + College/university | 1.75 | 1.03 -3.03 | 0.04 |
| **Study sites** |  |  |  |
| Village A | Ref |  |  |
| Village B | 3.22 | 1.74 – 6.04 | <0.001 |
| Village C | 3.56 | 1.90 – 6.78 | <0.001 |
| Village D | 1.89 | 1.01 – 3.57 | 0.05 |
| **Primary livelihood** |  |  |  |
| Crop production | Ref |  |  |
| Domestic animal related business | 0.47 | 0.13 – 1.65 | 0.24 |
| Homemaker | 0.71 | 0.38 – 1.31 | 0.27 |
| Non-animal related business | 0.98 | 0.54 – 1.77 | 0.94 |
| Unemployed/student/child | 0.71 | 0.28 – 1.78 | 0.47 |
| Wildlife related business | 0.64 | 0.17 – 2.13 | 0.47 |
| **Crowding index** | 0.93 | 0.58 – 1.51 | 0.78 |
| **Dedicated location for waste** | 0.97 | 0.62 – 1.50 | 0.88 |
| No | Ref |  |  |
| Yes | 1.86 | 0.86 – 4,12 | 0.11 |

*Missing values, if any, were omitted before the regression analysis process.

**Table S4. Treatment seeking behavior of the study participants**

|  | Village A (n = 154) | Village B (n = 112) | Village C  (n = 111) | Village D (n = 100) | Total (N = 477) | p-value Chi-square test |
| --- | --- | --- | --- | --- | --- | --- |
| Clinic / health centre | 100 (65%) | 107 (96%) | 107 (97%) | 93 (93%) | 407 (85%) | <0.001 |
| Dispensary / pharmacy | 57 (37%) | 90 (80%) | 96 (87%) | 38 (38%) | 281 (59%) | <0.001 |
| Community health worker | 148 (96%) | 89 (80%) | 61 (55%) | 87 (87%) | 385 (81%) | <0.001 |
| Mobile clinic | 1 (1%) | 1 (1%) | 0 (0%) | 0 (0%) | 2 (0%) | 1.000 |
| Hospital | 64 (42%) | 79 (71%) | 75 (68%) | 54 (54%) | 272 (57%) | <0.001 |
| Traditional healer | 37 (24%) | 0 (0%) | 1 (1%) | 1 (1%) | 39 (8%) | <0.001 |

**Table S5. Self-reported unusual symptoms in the past year period.**

|  | Village A (n = 154) | Village B (n = 112) | Village C  (n = 111) | Village D (n = 100) | Total (N = 477) | p value |
| --- | --- | --- | --- | --- | --- | --- |
| Symptoms_hemorrhagic fever | 6 (4%) | 0 (0%) | 2 (2%) | 4 (4%) | 12 (3%) | 0.147 |
| Symptoms_SARI^ | 60 (39%) | 16 (14%) | 11 (10%) | 24 (24%) | 111 (23%) | <0.001 |
| Symptoms_ILI* | 107 (70%) | 68 (61%) | 83 (75%) | 68 (68%) | 326 (68%) | 0.157 |
| Symptoms_enchepalitis | 110 (71%) | 92 (82%) | 86 (78%) | 68 (68%) | 356 (75%) | 0.067 |

^ SARI = Severe Acute Respiratory Infection, * ILI = Influenza like illness

**Table S6. Correlation between wildlife contacts and self-reported symptoms**

| **Hemorrhagic fever** | | | | |
| --- | --- | --- | --- | --- |
|  | Hemorrhagic fever – No  (n = 465) | Hemorrhagic fever – Yes  (n= 12) | Total  (n = 477) | P value / OR |
| Wildlife hunted/trapped |  |  |  | **0.0116 / 4.34** |
| No (N (Row%) | 417 (98%) | 8 (2%) | 425 (89%) |  |
| Yes (N (Row%) | 48 (93%) | 4 (8%) | 52 (11%) |  |
| Wildlife scratched/bitten |  |  |  | **0.0007 / 10.1** |
| No (N (Row%) | 456 (98%) | 10 (2%) | 466 (98%) |  |
| Yes (N (Row%) | 9 (82%) | 2 (18%) | 11 (2%) |  |
| Wildlife slaughtered |  |  |  | 0.120 / 3.24 |
| No (N (Row%) | 438 (98%) | 10 (2%) | 448 (94%) |  |
| Yes (N (Row%) | 27 (93%) | 2 (7%) | 29 (6%) |  |
| Wildlife cooked/handled |  |  |  | 0.616 / 1.40 |
| No (N (Row%) | 148 (98%) | 3 (2%) | 151 (32%) |  |
| Yes (N (Row%) | 317 (97%) | 9 (3%) | 326 (68%) |  |
| **SARI** | | | | |
|  | SARI – No  (n = 366) | SARI – Yes  (n= 111) | Total  (n = 477) | P value / OR |
| Wildlife hunted/trapped |  |  |  | **0.0165 / 2.08** |
| No (N (Row%) | 333 (78%) | 92 (21%) | 425 (89%) |  |
| Yes (N (Row%) | 33 (63%) | 19 (37%) | 52 (11%) |  |
| Wildlife scratched/bitten |  |  |  |  |
| No (N (Row%) | 358 (77%) | 108 (23%) | 466 (98%) | 0.751 / 1.24 |
| Yes (N (Row%) | 8 (73%) | 3 (27%) | 11 (2%) |  |
| Wildlife slaughtered |  |  |  | **0.00101 / 3.38** |
| No (N (Row%) | 351 (78%) | 97 (22%) | 448 (94%) |  |
| Yes (N (Row%) | 15 (52%) | 14 (48%) | 29 (6%) |  |
| Wildlife cooked/handled |  |  |  | 0.368 / 0.814 |
| No (N (Row%) | 112 (74%) | 39 (26%) | 151 (32%) |  |
| Yes (N (Row%) | 254 (78%) | 72 (22%) | 326 (68%) |  |
| **ILI** | | | | |
|  | ILI – No  (n = 151) | ILI – Yes  (n= 326) | Total  (n = 477) | P value / OR |
| Wildlife hunted/trapped |  |  |  | 0.2264 / 0.713 |
| No (N (Row%) | 131 (31%) | 294 (69%) | 425 (89%) |  |
| Yes (N (Row%) | 20 (38%) | 32 (62%) | 52 (11%) |  |
| Wildlife scratched/bitten |  |  |  | 0.320 / 0.548 |
| No (N (Row%) | 146 (31%) | 320 (69%) | 466 (98%) |  |
| Yes (N (Row%) | 5 (45%) | 6 (55%) | 11 (2%) |  |
| Wildlife slaughtered |  |  |  | **0.0165 / 0.407** |
| No (N (Row%) | 136 (30%) | 312 (70%) | 448 (94%) |  |
| Yes (N (Row%) | 15 (52%) | 14 (48%) | 29 (6%) |  |
| Wildlife cooked/handled |  |  |  | **0.0381 / 0.634** |
| No (N (Row%) | 38 (25%) | 113 (75%) | 151 (32%) |  |
| Yes (N (Row%) | 113 (35%) | 213 (65%) | 326 (68%) |  |
| **Encephalitis** | | | | |
|  | Encephalitis – No  (n = 121) | Encephalitis – Yes  (n= 356) | Total  (n = 477) | P value |
| Wildlife hunted/trapped |  |  |  | 0.949 / 1.02 |
| No (N (Row%) | 108 (25%) | 317 (75%) | 425 (89%) |  |
| Yes (N (Row%) | 13 (25%) | 39 (75%) | 52 (11%) |  |
| Wildlife scratched/bitten |  |  |  | 0.396 / 0.587 |
| No (N (Row%) | 117 (25%) | 349 (75%) | 466 (98%) |  |
| Yes (N (Row%) | 4 (36%) | 7 (64%) | 11 (2%) |  |
| Wildlife slaughtered |  |  |  | 0.777 / 0.885 |
| No (N (Row%) | 113 (25%) | 335 (75%) | 448 (94%) |  |
| Yes (N (Row%) | 8 (28%) | 21 (72%) | 29 (6%) |  |
| Wildlife cooked/handled |  |  |  | 0.0817 / 1.47 |
| No (N (Row%) | 46 (30%) | 105 (70%) | 151 (32%) |  |
| Yes (N (Row%) | 75 (23%) | 251 (77%) | 326 (68%) |  |
